# A cluster-randomized field trial to reduce cesarean section rates with a multifaceted intervention in Shanghai, China

**DOI:** 10.1186/s12916-020-1491-6

**Published:** 2020-02-14

**Authors:** Lulu Zhang, Lin Zhang, Meng Li, Jie Xi, Xiaohua Zhang, Zhenni Meng, Ying Wang, Huaping Li, Xiaohua Liu, Feihua Ju, Yuping Lu, Huijun Tang, Xianju Qin, Yanhong Ming, Rong Huang, Guohong Li, Hongying Dai, Rong Zhang, Min Qin, Liping Zhu, Jun Zhang

**Affiliations:** 1 Ministry of Education-Shanghai Key Laboratory of Children’s Environmental Health, Xinhua Hospital, Shanghai Jiao Tong University School of Medicine, Shanghai, China; 2 Department of Obstetrics and Gynecology, Xinhua Hospital, Shanghai Jiao Tong University School of Medicine, Shanghai, China; 3 Shanghai Jiao Tong University School of Public Health, Shanghai, China; 4 Department of Obstetrics, Jiading District Maternal and Child Health Hospital, Shanghai, China; 5 Department of Maternal Health Care, Minhang District Maternal and Child Health Hospital, Shanghai, China; 6 Department of Obstetrics, First Maternity and Infant Hospital, Tongji University School of Medicine, Shanghai, China; 7 Department of Obstetrics, Songjiang District Maternal and Child Health Hospital, Shanghai, China; 8 Department of Obstetrics and Gynecology, Sixth People’s Hospital, Shanghai Jiao Tong University School of Medicine, Shanghai, China; 9 Department of Obstetrics, China Welfare Association International Peace Maternal and Child Health Hospital, Shanghai Jiao Tong University School of Medicine, Shanghai, China; 10 Department of Obstetrics and Gynecology, Pudong New District Maternal and Child Health Hospital, Shanghai, China; 11 Department of Obstetrics and Gynecology, Pudong New Area People’s Hospital, Shanghai, China; 12 Department of Obstetrics, Putuo District Maternal and Child Health Hospital, Shanghai, China; 13 Department of General Surgery, Eighth People’s Hospital, Shanghai, China; 14 Center for HTA, China Hospital Development Institute, Shanghai Jiao Tong University, Shanghai, China; 15 Nursing College, Shanghai University of Medicine & Health Sciences, Shanghai, China; 16 Shanghai Maternal and Child Health Center, Shanghai, China

**Keywords:** Cesarean section, Health education, Intervention, Midwives, Randomized controlled trial

## Abstract

**Background:**

Cesarean section (CS) rate has risen dramatically and stayed at a very high level in China over the past two to three decades. Given the short- and long-term adverse effects of CS, effective strategies are needed to reduce unnecessary CS. We aimed to evaluate whether a multifaceted intervention would decrease the CS rate in China.

**Methods:**

We carried out a cluster-randomized field trial with a multifaceted intervention in Shanghai, China, from 2015 to 2017. A total of 20 hospitals were randomly allocated into an intervention or a control group. The intervention consisted of more targeted health education to pregnant women, improved hospital CS policy, and training of midwives/doulas for 8 months. The study included a baseline survey, the intervention, and an evaluation survey. The primary outcome was the changes of overall CS rate from the pre-intervention to the post-intervention period. A subgroup analysis stratified by the Robson classification was also conducted to examine the CS change among women with various obstetric characteristics.

**Results:**

A total of 10,752 deliveries were randomly selected from the pre-intervention period and 10,521 from the post-intervention period. The baseline CS rates were 42.5% and 41.5% in the intervention and control groups, respectively, while the post-intervention CS rates were 43.4% and 42.4%, respectively. Compared with the control group, the intervention did not significantly reduce the CS rate (adjusted OR = 0.92; 95% CI 0.73, 1.15). Similar results were obtained in subgroup analyses stratified by the risk level of pregnancy, maternal age, number of previous CS, or parity. Scarred uterus and maternal request remained the primary reasons for CS after the interventions in both groups. The intervention did not alter the perinatal outcomes (adjusted change of risk score = − 0.06; 95%CI − 0.43, 0.31).

**Conclusions:**

A multifaceted intervention including more targeted prenatal health education, improved hospital CS policy, and training of midwives/doulas, did not significantly reduce the CS rate in Shanghai, China. However, our experience in implementing a multifaceted intervention may provide useful information to other similar areas with high CS use.

**Trial registration:**

This trial was registered at the Chinese Clinical Trial Registry (www.chictr.org.cn) (ChiCTR-IOR-16009041) on 17 August 2016.

## Introduction

Cesarean section (CS) rate has increased dramatically in many parts of the world in the last few decades [1]. It was estimated that the average CS rate worldwide increased from 12.1% in 2000 to 21.1% in 2015 with an average annual rate of increase of 3.7% [1]. The CS rate in China began to rise in the early 1980s, with a sharp rise in the mid-1990s, and continued to rise from 28.8% in 2008 to 34.9% in 2014 [2, 3]. Reasons for the rapid rise in CS rate in China were multifactorial [4–6]. Among the commonly cited reasons are fear of labor pain, concerns about complications such as urinary incontinence and lower quality of sex life after vaginal delivery, misconception of CS being safer than vaginal delivery for the baby, poor experience of previous vaginal delivery, and auspicious dates [3, 4]. The shortage of nurses/midwives and the large volume of deliveries often lead to more convenient and scheduled CS. The constrained doctor-patient relationship and insufficient training in vaginal delivery also exacerbated the situation [5, 7]. Higher financial incentives for CS versus vaginal delivery may lead to the preferred choice of CS [2, 4, 5].

Extensive evidence has shown that CS without medical indications is associated with an increased risk of short-term and long-term adverse outcomes as well as substantial economic burden [8–10]. The downside of widespread CS is now fully manifested in China where the government changed to a two-child policy recently. A high proportion of multiparous women have a scarred uterus, abnormal placenta implantation, and repeated CS [11, 12].

Shanghai is one of the largest cities in China. The CS rate increased from 17.5% in the early 1980s to 55% in 2010 [13, 14]. Despite that the rate has declined to 47.9% in 2016, it remains high [15]. Given that the causes of the high CS rate are multifactorial, previous studies suggested that multifaceted interventions be used to decrease the CS rate [5, 6, 16–22]. However, evidence concerning effective approaches to reduce unnecessary CS is limited [23, 24], especially in China and other low- and mid-income countries. Therefore, we conducted a cluster-randomized field trial in Shanghai, China, where the CS rate is very high [2, 17, 25], to examine the effects of a multifaceted strategy targeting mothers, health professionals, and hospital policy to reduce the CS rate.

## Methods

### Study design and hospitals

This stratified, cluster-randomized, parallel-group field trial was conducted to examine the effects of a multifaceted intervention on the use of CS in 20 hospitals in Shanghai, China, from 2015 to 2017. Hospitals were invited to participate in this trial and were informed that they could be assigned to either intervention or control group. Seven tertiary hospitals and 13 secondary hospitals agreed to participate. Primary care hospitals were not included in the present study, as they usually do not provide obstetric services, and very few women are delivered outside of the hospital. The participating hospitals delivered approximately half of all births in Shanghai (approximately 200,000 births per year).

An ethical approval was obtained from the Ethics Review Board of the Xinhua hospital, Shanghai Jiao Tong University School of Medicine and other participating hospitals (Approval number: XHEC-C-2016-095).

### Randomization and masking

Hospitals were first stratified by their levels (tertiary vs secondary). Within each stratum, hospitals were randomly assigned to the intervention and control groups and designated as the units of randomization to ensure that there would be minimal cross contamination between the intervention and control groups. The randomization was conducted by the data management group. The study included a 6-month pre-intervention (baseline survey) period, an 8-month intervention period, and a 6-month post-intervention (evaluation survey) period. No masking was applied in this study.

### Baseline survey

Before the intervention, we conducted a baseline survey. A total of 62,653 births were delivered in the 20 hospitals from January 1 to June 30 in 2016. A random sample of all births was selected. To ensure the precision of the CS rate estimates, we randomly selected 20% of the total births in hospitals with an annual delivery volume under 10,000, and 10% in hospitals with an annual delivery volume over 10,000 [26]. On average, about 500 records were extracted per hospital. To make our findings comparable to other studies, we further restricted the analysis to women whose newborns had a gestational age of at least 24 weeks or weighed at least 500 g at delivery. Finally, a total of 10,807 deliveries remained to represent the total births in these hospitals during that period. Medical records of mothers and newborns were retrieved, and information on maternal demographic characteristics, reproductive history, and maternal and neonatal conditions were abstracted by specially trained research staff for both the baseline and evaluation surveys.

### Interventions

A multifaceted intervention was developed based on previous research [5, 22, 27]. It consisted of three components. First, a targeted health education program on top of the regular prenatal education was developed to familiarize pregnant women with the process of natural childbirth, and the health benefits and risks of CS. Educational brochures, 15 online and offline courses, and free outpatient consultations were offered to women free of charge in the intervention hospitals. These education programs covered the whole pregnancy from booking to delivery. A set of brochures on various topics of CS and natural birth were developed. A number of prenatal health courses were recorded and placed online for women to view at any time. Some talks by health professionals were broadcasted live. In-person classes were also held at weekends for women to attend free of charge. The list of courses was described in more detail in Additional file 1.

Second, after a careful review of the literature, a focus group discussion with obstetricians, midwives, and hospital administers, and consultation with hospital management, an improved hospital CS policy was established and promoted in the intervention hospitals. The policy included three measures: To install a CS second opinion process, i.e., if an obstetrician decides to perform a CS on a woman, he/she needs to request a review by the unit chief or a designated senior physician for a second opinion [16, 27, 28]. The obstetric departments in the intervention hospitals were also encouraged to conduct regular peer reviews of CS indications, post the monthly CS rate, and implement a reward system [16, 27–31].

Third, several training courses with a specially designed syllabus were offered to midwives and doulas in the intervention hospital to improve their skills.

The intervention was implemented from September 1, 2016, to April 30, 2017. The control group did not receive any of the above interventions except for providing the usual care.

### Evaluation survey

A total of 54,257 births were delivered in the participating hospitals during the evaluation period from May 1 to October 31 in 2017. A random sample was selected, and data were abstracted in the same way as that of the baseline survey. A total of 10,553 deliveries remained for analyses (Additional files 2, 3, and 4).

### Assessment of outcomes

The main outcomes were at the individual participant level within hospitals (randomization units). The primary outcome was the changes of the overall CS rate from the pre-intervention to the post-intervention period. A subgroup analysis stratified by the Robson classification was also conducted to examine the CS change among women with various obstetric characteristics. The secondary outcomes were gestational weight gain (GWG), obstetrical interventions, and perinatal outcomes. GWG was defined as the difference between documented weight at the first and last prenatal visit just before delivery. Obstetrical interventions included planned and intrapartum CS rate, artificial rupture of the membranes, labor induction, oxytocin use during labor, epidural analgesia, use of doula, assisted vaginal delivery, and episiotomy. Planned and intrapartum CS were defined as CS before and after the onset of labor, respectively. Labor induction was defined as artificially induced uterine contraction. Perinatal outcomes were measured by a composite score, defined by the American College of Obstetricians and Gynecologists (ACOG) Quality Improvement and Patient Safety Committee (QuIPS) [32]. Each of the 10 outcomes was assigned a weighted score indicating the severity: maternal death, 750 points; intrapartum or neonatal death > 2500 g, 400 points; uterine rupture, 100 points; maternal admission to ICU, 65 points; birth trauma, 60 points; return to operating room/labor and delivery, 40 points; admission to NICU > 2500 g and for > 24 h, 35 points; APGAR < 7 at 5 min, 25 points; blood transfusion, 20 points; and 3°- or 4°-perineal tear, 5 points. The individual perinatal risk score was computed by the sum of the scores of all the 10 outcomes (if any) to manifest perinatal outcome for each mother.

### Assessment of covariates

Maternal age was treated as a continuous variable. Information on maternal height, weight at delivery, nulliparous (yes/no), assisted reproductive technology (ART; yes/ no), previous cesarean delivery (yes/no), gestational age (GA) at delivery, birthweight of newborn, pathology (yes/no), and hospital level (tertiary hospitals/secondary hospitals) were collected. Body mass index (BMI) was calculated as maternal weight at delivery in kilograms divided by the square of height in meters (kg/m^2^).

A pregnancy was considered as morbid if any of the following conditions was met: non-cephalic presentation of the fetus, placental abruption, placenta previa, uterine rupture, hypertensive disorders of pregnancy (including gestational hypertension, preeclampsia, eclampsia, and HELLP syndrome), heart disease, deep venous thrombosis, kidney disease, pre-gestational and gestational diabetes mellitus, pre-gestational and gestational thyroid disease (including hyperthyroidism, hypothyroidism and others), premature rupture of membranes (gestational age < 37 weeks), Rh incompatibility, or congenital malformation.

A pregnancy was considered as low-risk if the newborn was born in cephalic presentation, and the mothers were aged 18 or above and younger than 40 years old, gave a term birth (37–41 completed weeks of gestation), had a pre-pregnancy BMI between 17 and 28 kg/m^2^, and were without previous ART, CS, prior or current stillbirth, and morbidity during pregnancy defined above. In contrast, a pregnancy was considered as high-risk with any of the above conditions.

### Statistical analysis

Based on the overall CS rate of 45% in Shanghai, we estimated that the intervention may reduce the CS rate by 7 to 38%. Assuming that the unit size was about 500 subjects per hospital and the intraclass correlation coefficient was 0.011, we calculated that we would need to enroll 20 hospitals for the purpose of the study to have 90% power to detect 7% reduction in CS rate. A two-sided alpha significance level of 0.05 was used.

Continuous variables were described as mean (standard deviation), whereas categorical variables were presented as numbers and percentages. An intention-to-treat analysis was applied according to the assignment of randomization at the beginning of the study. Given the clustering of women (final analysis units) within hospitals (randomization units), generalized estimating equations (GEE) were used to assess the effects of the multifaceted intervention on CS rates, GWG, obstetrical interventions, and perinatal outcomes, separately, adjusting for maternal age, BMI at delivery, parity, ART, previous CS history, GA at delivery, birthweight of the newborn, pregnancy complications, and hospital level. Adjusted OR, adjusted *β*, and corresponding 95% CIs were computed to compare the changes between the intervention group and the control group from the pre- to the post-intervention period. For the GEE models that did not converge, a logistic regression model was used with *p* values of less than 0.001 being considered as statistically significant and *p* values of less than 0.003 being marginally significant [33, 34].

We conducted stratified analyses by the risk level of the mothers (low vs. high), maternal age (≥ 35 vs. < 35), the number of previous CS (0 vs. ≥1), and parity (primipara vs. multipara without a previous CS vs. multipara with a previous CS or not). Additionally, to identify factors that were negatively associated with CS, we evaluated the intervention effect in each group of mothers by the modified Robson Classification System [35]. Five basic obstetric characteristics were used by the modified Robson classification system to categorize all subjects admitted for delivery: parity (nulliparous, multiparous with or without a previous CS), onset of labor (spontaneous labor, induced labor, or CS before labor), gestational age (preterm birth or full term), fetal presentation (cephalic, breech, transverse or oblique lie), and number of fetus (singleton or multiplets). In order not to miss significant information for the success of induction and its contribution to the CS rate, the modified Robson classification divided induced labor and CS before labor into two groups for nulliparous and multiparous women, respectively (groups 2, 3, 5, and 6). On the other hand, the number of women with transverse or oblique fetal lie was small but the CS rates for non-cephalic presentations were very high. Thus, these groups were combined into one (group 8). Moreover, subjects who lacked at least one of the above five obstetric characteristics were placed in the unknown group (group 99). After the appropriate expansion and reduction in certain categories, the total number of groups remained at 10, plus the unknown group. Specifically, group 1 (nulliparous, spontaneous: abbreviated as NS): nulliparous women with singleton cephalic pregnancy, ≥ 37 weeks in spontaneous labor; group 2 (nulliparous, induced: NI): nulliparous women with singleton cephalic pregnancy, ≥ 37 weeks in induced labor; group 3 (nulliparous, cesarean: NC): nulliparous women with singleton cephalic pregnancy, ≥ 37 weeks who were delivered by CS before labor; group 4 (multiparous, spontaneous: MS): multiparous women without a previous CS, with singleton cephalic pregnancy, ≥ 37 weeks in spontaneous labor; group 5 (multiparous, induced: MI): multiparous women without a previous CS, with singleton cephalic pregnancy, ≥ 37 weeks in induced labor; group 6 (multiparous, cesarean: MC): multiparous women without a previous CS, with singleton cephalic pregnancy, ≥ 37 weeks who were delivered by CS before labor; group 7 (previous cesarean: PC): multiparous women with a previous CS, with singleton cephalic pregnancy, ≥ 37 weeks; group 8 (breech: BR): all women with a singleton pregnancy with a breech, transverse, or oblique lie; group 9 (twin: TW): all women with multiple pregnancies (twins or higher-order multiples); group 10 (preterm: PT): all women with a singleton cephalic pregnancy, <  37 weeks [35].

All statistical analyses were conducted with SAS 9.4 software (SAS Institute Inc., Cary, NC). This trial was registered at the Chinese Clinical Trial Registry (www.chictr.org.cn) (ChiCTR-IOR-16009041).

## Results

**Fig. 1:**
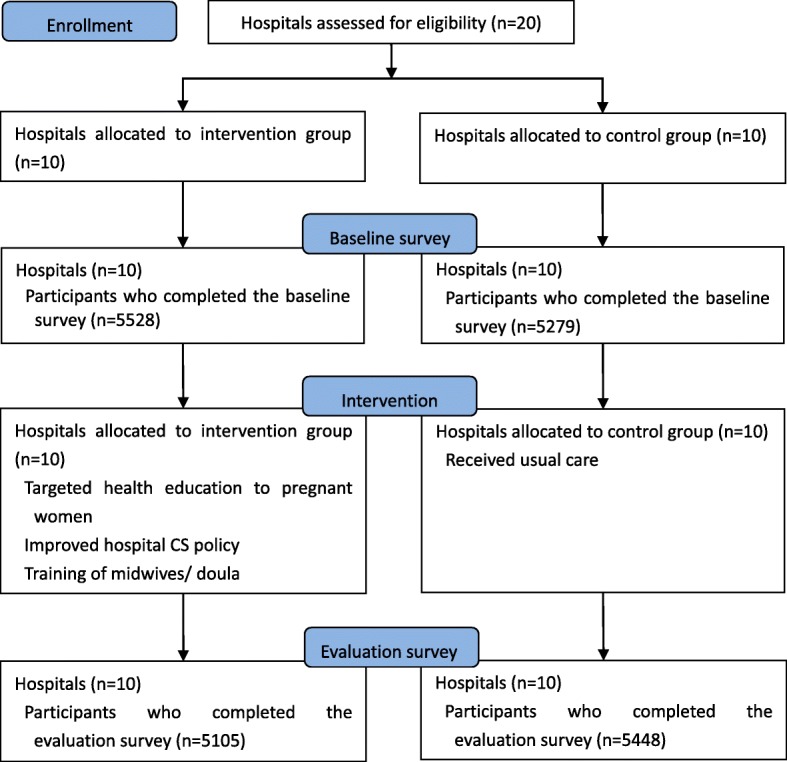
Trial profile

The intervention group consisted of three tertiary and seven secondary hospitals, and the control group was composed of four tertiary and six secondary hospitals. From January 1, 2016, to October 31, 2017, a total of 21,360 deliveries were randomly sampled during the study period, including 10,807 deliveries in the pre-intervention and 10,553 in the post-intervention period (Fig. 1). After excluding women with missing information on labor and delivery, 21,273 women (99.6%) were included in the final analysis. Baseline characteristics including maternal age, parity, GA at delivery, CS history, risk level of the pregnancy, birth outcome, and neonatal birthweight were generally similar between the intervention and control groups. In contrast, newborns were slightly more likely to be non-cephalic presentation in the intervention group than in the control group (5.3% vs. 4.3%) (Table 1).

**Table 1:** Characteristics of hospitals and patients by group allocation during the baseline period, Shanghai, China

	Intervention Group (*N* = 5498)	Control group (*N* = 5254)
Hospitals		
Type of hospital, no. (%)		
Tertiary hospitals	1941 (35.3)	1813 (34.5)
Secondary hospitals	3557 (64.7)	3441 (65.5)
Patients		
Maternal age at delivery, years		
< 18 years, no. (%)	12 (0.2)	19 (0.4)
18–34 years, no. (%)	4842 (89.9)	4686 (90.1)
≥ 35 years, no. (%)	533 (9.9)	494 (9.5)
Missing	111	55
Pre-pregnancy body mass index, kg/m^2^	22.0 ± 3.2	22.3 ± 3.3
Parity, no. (%)		
0	3546 (64.5)	3399 (64.7)
1	1828 (33.3)	1715 (32.7)
≥ 2	121 (2.2)	137 (2.6)
Missing	3	3
Gestational age at delivery, no. (%)		
< 37 weeks	312 (5.7)	292 (5.6)
37–41 weeks	5167 (94.0)	4948 (94.2)
≥ 42 weeks	19 (0.4)	14 (0.3)
Previous cesarean deliveries, no. (%)		
No	4722 (86.0)	4516 (86.1)
Yes	768 (14.0)	727 (13.9)
Missing	8	11
Risk level of pregnancy, no. (%)		
Low	2830 (51.5)	2671 (50.8)
High	2668 (48.5)	2583 (49.2)
Presentation of the baby		
Cephalic	5088 (94.7)	4964 (95.7)
Breech/transverse/oblique lie	284 (5.3)	222 (4.3)
Missing	126	68
Mode of delivery		
Vaginal delivery	3034 (55.2)	3003 (57.2)
Assisted vaginal delivery	128 (2.3)	70 (1.3)
Planned cesarean delivery	2000 (36.4)	1855 (35.3)
Intrapartum cesarean delivery	336 (6.1)	326 (6.2)
Birth outcome		
Live birth	5479 (99.7)	5236 (99.7)
Fetal death	14 (0.3)	13 (0.3)
Stillbirth	1 (0.02)	2 (0.04)
Neonatal death	4 (0.07)	3 (0.06)
Neonatal birthweight, g		
< 1500, no. (%)	16 (0.3)	17 (0.3)
1500–2499, no. (%)	185 (3.4)	191 (3.6)
2500–3999, no. (%)	4901 (89.2)	4663 (88.8)
≥ 4000, no. (%)	395 (7.2)	383 (7.3)
Missing	1	0

Outcome: twins combined

Neonatal admission to ICU: twins combined

The baseline CS rates were 42.5% and 41.5% in the intervention and control groups, respectively, versus 43.4% and 42.4% in the post-intervention period, respectively. The intervention did not significantly reduce the CS rate when comparing with the control group during the same period (adjusted OR = 0.92; 95%CI 0.73, 1.15; *p* = 0.44) (Table 2). Similar results were obtained after we stratified the women by the risk level of the pregnancy, maternal age, number of previous CS, or parity. Among the low-risk women, there was a small reduction in CS rate in both groups from the baseline to the post-intervention periods (− 3.6% and − 2.8%, respectively). In contrast, the CS rates in both groups increased during the same period in high-risk pregnancies (2.5% and 4.0%, respectively). Although none of the subgroup analyses showed any statistically significant reduction, all the point estimates of the adjusted odds ratios were below 1.

**Table 2:** Effects of the multifaceted intervention on cesarean section rate, Shanghai, China

	Intervention group (10 hospitals)				Control group (10 hospitals)				Effect of the intervention	
	Baseline (*N* = 5498)	Evaluation (*N* = 5092)	Rate change%	*p* value	Baseline (*N* = 5254)	Evaluation (*N* = 5429)	Rate change%	*p* value	Adjusted OR (95%CI)	*p* value
Cesarean delivery, no. (%)	2336 (42.5)	2209 (43.4)	0.9	0.32	2181 (41.5)	2303 (42.4)	0.9	0.47	0.92 (0.73, 1.15)	0.44
Risk level of pregnancy										
Low										
Total no.	2830	2420			2671	2711				
Cesarean delivery, no. (%)	760 (26.9)	563 (23.3)	−3.6	0.20	691 (25.9)	626 (23.1)	−2.8	0.36	0.96 (0.74, 1.25)	0.75
High										
Total no.	2668	2672			2583	2718				
Cesarean delivery, no. (%)	1576 (59.1)	1646 (61.6)	2.5	0.97	1490 (57.7)	1677 (61.7)	4.0	0.14	0.9 (0.71, 1.14)	0.38
Maternal age										
< 35 years										
Total no.	4854	4330			4705	4605				
Cesarean delivery, no. (%)	1969 (40.6)	1756 (40.6)	0	0.25	1879 (39.9)	1847 (40.1)	0.2	0.63	0.9 (0.71, 1.13)	0.36
≥ 35 years										
Total no.	533	678			494	739				
Cesarean delivery, no. (%)	317 (59.5)	413 (60.9)	1.4	0.74	283 (57.3)	418 (56.6)	−0.7	0.51	0.97 (0.58, 1.62)	0.89
Number of previous cesarean deliveries										
0										
Total no.	4722	4134			4516	4496				
Cesarean delivery, no. (%)	1594 (33.8)	1289 (31.2)	−2.6	0.32	1489 (33.0)	1403 (31.2)	−1.8	0.73	0.94 (0.74, 1.19)	0.59
≥ 1										
Total no.	768	952			727	930				
Cesarean delivery, no. (%)	739 (96.2)	920 (96.6)	0.4	0.95	687 (94.5)	900 (96.8)	2.3	0.06	0.55 (0.25, 1.24)	0.14**
Parity										
Primipara										
Total no.	3546	2837			3399	3064				
Cesarean delivery, no. (%)	1405(39.6)	1094 (38.6)	−1.0	0.56	1316 (38.7)	1172 (38.3)	−0.4	0.86	0.98 (0.77, 1.25)	0.87
Multipara without a previous CS										
Total no.	1175	1298			1117	1432				
Cesarean delivery, no. (%)	188 (16.0)	195 (15.0)	−1.0	0.24	173 (15.5)	231 (16.1)	0.6	0.15	0.74 (0.49, 1.1)	0.13
Multipara with a previous CS or not										
Total no.	1949	2255			1852	2364				
Cesarean delivery, no. (%)	929 (47.7)	1115 (49.5)	1.8	0.25	863 (46.6)	1131 (47.8)	1.2	0.045	0.75 (0.53, 1.05)	0.09

Intention-to-treat analysis

**A logistic model was used because the calculations for the GEE model did not converge. For this model, *p* values of less than 0.001 were considered to indicate statistical significance and *p* values of less than 0.003 were considered to indicate marginal significance

Table 3 shows the CS rate by the categories of the modified Robson Classification System and by group allocation and period. Similarly, the intervention did not significantly affect the CS rates across the Robson categories.

**Table 3:** Cesarean section rates by obstetric characteristics according to the modified Robson classification by group allocation and period, Shanghai, China

	Intervention group (10 hospitals)			Control group (10 hospitals)			Effect estimates	
Cesarean by indications (modified Robson classification)	Baseline (*N* = 5498)	Evaluation (*N* = 5092)	Rate change%	Baseline (*N* = 5254)	Evaluation (*N* = 5429)	Rate change%	Adjusted OR (95%CI)	*p* value
1. Nulliparous, singleton, cephalic, ≥ 37 weeks, spontaneous labor								
Total no.	1586	1100		1619	1259			
Cs, no.(%)	129 (8.1)	89 (8.1)	0	160 (9.9)	101 (8.0)	−1.9	1.24 (0.71, 2.17)	0.44
2. Nulliparous, singleton, cephalic, ≥ 37 weeks, induced labor								
Total no.	717	761		660	805			
Cs, no.(%)	160 (22.3)	132 (17.4)	−4.9	141 (21.4)	148 (18.4)	−3.0	0.87 (0.52, 1.46)	0.60
3. Nulliparous, singleton, cephalic, ≥ 37 weeks, CS before labor								
Total no.	819	650		762	714			
Cs, no.(%)	819 (100)	650 (100)	0	762 (100)	714 (100)	0	NA	NA
4. Multiparous, singleton, cephalic, ≥ 37 weeks, without a previous CS, spontaneous labor								
Total no.	796	764		753	886			
Cs, no.(%)	22 (2.8)	11 (1.4)	−1.4	13 (1.7)	10 (1.1)	−0.6	0.85 (0.27, 2.72)	0.78**
5. Multiparous, singleton, cephalic, ≥ 37 weeks, without a previous CS, induced labor								
Total no.	159	329		166	294			
Cs, no.(%)	11 (6.9)	21 (6.4)	−0.5	11 (6.6)	14 (4.8)	−1.8	1.03 (0.32, 3.35)	0.96**
6. Multiparous, singleton, cephalic, ≥ 37 weeks, without a previous CS, CS before labor								
Total no.	97	103		96	136			
Cs, no.(%)	97 (100)	103 (100)	0	96 (100)	136 (100)	0	NA	NA
7. Multiparous, singleton, cephalic, ≥ 37 weeks, with a previous CS								
Total no.	688	853		664	842			
Cs, no.(%)	666 (96.8)	827 (97.0)	0.2	631 (95.0)	818 (97.2)	2.2	0.52 (0.21, 1.29)	0.15**
8. Singleton, breech, transverse or oblique lie^#^								
Total no.	284	230		222	206			
Cs, no.(%)	237 (83.5)	202 (87.8)	4.3	213 (96.0)	197 (95.6)	−0.4	2.05 (0.4, 10.65)	0.39
9. Multiple pregnancy (twins or higher-order multiples)								
Total no.	113	90		66	64			
Cs, no.(%)	108 (95.6)	84 (93.3)	−2.3	58 (87.9)	59 (92.2)	4.3	0.25 (0.02, 3.25)	0.28**
10. Singleton, cephalic, < 37 weeks								
Total no.	217	201		231	217			
Cs, no.(%)	80 (36.9)	88 (43.8)	6.9	91 (39.4)	107 (49.3)	9.9	0.7 (0.34, 1.43)	0.32
99. Unknown								
Total no.	22	11		15	6			
Cs, no.(%)	7 (31.8)	2 (18.2)	−13.6	5 (33.3)	1 (16.7)	−16.6	NA	NA

**A logistic model was used because the calculations for the GEE model did not converge. For this model, *p* values of less than 0.001 were considered to indicate statistical significance and *p* values of less than 0.003 were considered to indicate marginal significance

#The number of subjects in transverse or oblique lie was small while the CS rates for non-cephalic presentations were very high; thus, these groups were combined into one

Abbreviation: *NA* not applicable

The baseline GWG were 13.2 (SD 5.3) kg and 12.3 (SD 5.5) kg in the intervention and control groups, respectively, versus 11.2 (SD 4.8) kg and 11.2 (SD 5.1) kg in the post-intervention period, respectively. The intervention did not significantly reduce the GWG when comparing with the control group during the same period (adjusted *β* = − 0.05; 95%CI − 0.11, 0.01; *p* = 0.11). Scarred uterus, CS by maternal request without medical indication, abnormal fetal heart rate pattern, breech or transverse presentation, prolonged labor, and macrosomia ranked the top six primary indications for CS in both groups during the baseline and the evaluation periods. The proportion of CS due to scarred uterus had increased from the baseline to the evaluation period in both groups (4.6% and 3.6%, respectively) (Table 4). The frequencies of obstetrical interventions were similar between the two groups before and after the intervention (Table 5). However, among women who had a trial of labor, the intervention appeared to have had a suggestive but statistically non-significant effect on assisted vaginal delivery (OR = 0.61, 95%CI 0.31, 1.21) (Table 5).

**Table 4:** Hospital-based cesarean section rates by the primary indication and by the group allocation during the baseline and post-intervention periods, Shanghai, China

	Intervention group (10 hospitals)		Control group (10 hospitals)		*p* value
Cesarean indications	Baseline (*N* = 5498)	Evaluation (*N* = 5092)	Baseline (*N* = 5254)	Evaluation (*N* = 5429)	
Scarred uterus, no. (%)	716 (13.0)	897 (17.6)	632 (12.3)	863 (15.9)	0.24
Cesarean section without medical indication, no. (%)	480 (8.7)	401 (7.9)	456 (8.7)	429 (7.9)	0.21
Abnormal fetal heart rate (FHR) pattern, no. (%)	325 (5.9)	257 (5.1)	413 (7.9)	395 (7.3)	0.08
Breech or transverse presentation, no. (%)	230 (4.2)	173 (3.4)	200 (3.8)	178 (3.3)	0.24
Macrosomia, no. (%)	135 (2.5)	106 (2.1)	109 (2.1)	92 (1.7)	0.71
Prolonged labor, no. (%)	65 (1.2)	69 (1.4)	49 (0.9)	39 (0.7)	0.30
Other indications, no. (%)	382 (7.0)	303 (6.0)	317 (6.0)	300 (5.5)	0.11
Total, no. (%)	2333 (42.4)	2206 (43.3)	2176 (41.4)	2296 (42.3)	0.01

**Table 5:** Obstetrical intervention rates by group allocation and period, and their impact on cesarean section rate

	Intervention group (10 hospitals)				Control group (10 hospitals)				Effect estimates	
Obstetrical interventions	Baseline (*N* = 5498)	Evaluation (*N* = 5092)	Rate change%	*p* value	Baseline (*N* = 5254)	Evaluation (*N* = 5429)	Rate change%	*p* value	Adjusted OR (95%CI)	*p* value
All deliveries										
Women without a previous CS										
Planned cesarean delivery, no. (%)	1287 (27.3)	1067 (25.8)	−1.5	0.85	1177 (26.1)	1169 (26.0)	−0.1	0.07	0.9 (0.73, 1.1)	0.30
Women with a previous CS or not										
Planned cesarean delivery, no. (%)	2000 (36.4)	1946 (38.2)	1.8	0.72	1855 (35.3)	2049 (37.7)	2.4	0.03	0.85 (0.69, 1.05)	0.13
Labor induction, no. (%)	1050 (19.1)	1282 (25.2)	6.1	< 0.0001	1010 (19.3)	1347 (24.9)	5.6	< 0.0001	1.07 (0.77, 1.5)	0.67
Women who attempted labor										
Total no.	3498	3146			3399	3380				
Women without a previous CS										
Intrapartum cesarean delivery, no. (%)	307 (8.9)	222 (7.2)	−1.7	0.08	312 (9.3)	234 (7.0)	−2.3	0.02	1.07 (0.69, 1.64)	0.76
Women with a previous CS or not										
Intrapartum cesarean delivery, no. (%)	336 (9.6)	263 (8.4)	−1.2	0.10	326 (9.6)	254 (7.5)	−2.1	0.03	1.08 (0.74, 1.98)	0.68
Oxytocin during labor, no. (%)	529 (15.1)	542 (17.3)	2.2	< 0.0001	594 (17.5)	622 (18.5)	1.0	0.31	1.32 (0.81, 2.16)	0.26
Epidural analgesia, no. (%)	1135 (32.6)	959 (30.6)	−2.0	0.002	595 (17.6)	837 (25.7)	8.1	< 0.0001	0.61 (0.20, 1.81)	0.36
Doula, no. (%)	1315 (37.6)	1422 (45.3)	7.7	< 0.0001	1051 (34.7)	1115 (37.0)	2.3	0.27	1.35 (0.88, 2.08)	0.17
Women without a previous CS										
Assisted vaginal delivery, no. (%)	127 (3.7)	102 (3.3)	−0.4	0.77	69 (2.1)	99 (3.0)	0.9	0.03	0.61 (0.31, 1.21)	0.15
Women with a previous CS or not										
Assisted vaginal delivery, no. (%)	128 (3.7)	103 (3.3)	−0.4	0.77	70 (2.1)	103 (3.1)	1.0	0.03	0.6 (0.31, 1.18)	0.13
Episiotomy, no. (%)										
Median/lateral	1289 (36.9)	1006 (32.1)	−4.8	< 0.0001	1532 (45.1)	1374 (40.7)	−4.4	0.69	0.78 (0.46, 1.33)	0.36

Few women had severe complications. The proportion of women with an individual perinatal risk score defined by ACOG QuIPS above zero remained virtually the same from the baseline to the evaluation period in both groups (− 0.3% vs. − 0.2%, respectively).

## Discussion

This trial showed that a multifaceted intervention did not reduce the high CS rate in Shanghai, China. Scarred uterus and maternal request were still the primary indications for CS even after the intervention. To our best knowledge, our study was so far the first randomized trial to reduce the CS rate by a multifaceted intervention in China.

Numerous attempts have been made to reduce the CS rate around the world [27, 28, 34, 36–38]. Unfortunately, the impact of a single intervention approach has been inconsistent and mostly limited [39]. Chaillet et al. used multifaceted interventions, including audits of indications for CS, provision of feedback to health professionals, and implementation of best practices, and reported a statistically significant but small reduction in the CS rate (adjusted risk difference = − 1.8%; 95% CI − 3.8%, − 0.2%) [34]. A trial by Althabe et al. showed that a hospital policy of mandatory second opinion had a similar statistically significance but marginal reduction in CS use (adjusted risk difference = − 1.9%; 95% CI − 3.8%, − 0.1%) [27].

On the other hand, two Chinese retrospective observational studies suggested that multifaceted interventions involving government policy, finical incentives, local benchmarking, health education for health professionals and pregnant women, doula care, and access to labor analgesia could decrease the CS use effectively [40, 41]. In a retrospective study at a large maternity hospital in Shanghai, Liu et al. compared CS rates before and after the implementation of a multifaceted intervention [41], which included government and hospital measures. The government measure consisted of fixing per-patient reimbursement by the government health insurance regardless of the delivery mode, and ranking the obstetric departments by the CS rate. The hospital measure included free perinatal healthcare classes, improving women’s childbirth experience by allowing family and an experienced midwife to stay with them during labor and offering labor analgesia, ranking physicians’ performance within the hospital by CS rate. After the interventions, there was a 31% reduction in the CS rate, with an OR of 0.69 [95% CI 0.66–0.71]. However, the study did not separate the effects of the government and hospital measures.

Yu et al. conducted a retrospective pre-/post-intervention study which focused on CS on maternal request changed by institutional interventions and government policy [40]. Institutional interventions consisted of three aspects: providing health education to mothers and their families; training obstetricians and midwives, issuing CS indications and guidelines, and conducting audits every month; promoting labor analgesia and doula care by midwives. These interventions were similar to ours. In addition, the central and local government policy directly addressed the financial and management aspects. The overall CS rate declined by 1.3% and 8.3% attributable to the institutional and government interventions, respectively. Nonetheless, the previous studies in China were retrospective observational data analyses. The true impact needs to be evaluated in a randomized controlled fashion.

Despite that these studies were conducted in quite different settings and cultures, and all of them showed some effects of the multifaceted interventions, they seemed to have some common characteristics. First, studies with interventions initiated by academic organizations reduced CS rate only to a modest degree [24, 27, 34]. Instead, the interventions initiated by the maternity hospitals themselves showed a larger reduction in CS use [36, 42, 43]. Furthermore, the government-led efforts were more effective when the CS rate was included as a benchmark for hospital performance [40, 41]. For example, a nationwide intervention strategy in Portugal even reversed the national upward trend of the CS rate [38]. It was worth noting that the two studies in China evaluated the changes of the CS rate around 2012, when WHO published a report on the high CS rate, especially for those without medical indications in China. After the WHO report, the Chinese government became increasingly concerned of the adverse health impacts of the high CS rate and took a series of measures to address this issue [44].

It is also understandable that interventions conducted in a single hospital tended to gain more support from medical opinion leaders and, thus, easier to form a concerted action in the hospital. Similarly, the government-led interventions could be directly implemented in healthcare systems. In contrast, interventions initiated by academic researchers did not have such advantages. Kingdon et al. and Chaillet et al. found that the negotiation of health professionals with healthcare system and the practice environment including unit leadership, policy, availability of equipment, and the extent to undertake the guideline recommendation were the major keys to decrease the CS rate successfully [22, 45].

Due to a high CS rate for over a decade, a higher proportion of women in China have a scarred uterus comparing to other countries. Women with a previous CS were more likely to select repeat CS. This explains why our CS rate actually increased from the baseline to the post-invention period because during our trial, the two-child policy was instituted. The proportion of multiparas increased, many of whom had a previous CS, leading to a higher overall CS rate. However, in nulliparas, the CS rates did decline but the difference between the intervention and control groups did not reach statistical significance. It should be noted that the considerable flux of declining primary CS and increasing repeat CS may hamper a substantial decline in the overall CS rate in the near future.

Our study has several limitations. First, although we provided women more targeted health education via the online and offline programs free of charge in the intervention hospitals, approximately half of the women in the intervention hospitals participated in our targeted health education program. Thus, the overall impact may have been diluted. Second, due to a high volume of deliveries, it is challenging to provide one-on-one doula support throughout labor in most hospitals. A total of 30–40% had doula support, among whom some women shared a doula [46]. Third, despite our effort in promoting the improved hospital CS policy, the degree of adaptation varied by hospitals. Fourth, the high CS rate in Shanghai is mainly contributed by the high prelabor CS rate, which was our main target. Two of the three intervention components, namely health education and hospital CS policy, were designed to tackle this issue, particularly in nulliparous women. However, it takes time to change the culture and for the education to take effect. The duration of our intervention may be too short to see a significant impact. The impact might become statistically significant if the interventions continued for a longer period. Finally, our intervention package included three measures. It is difficult to disentangle their effects.

CS issue is extremely complex and deeply rooted. Despite our recognition of their importance, some measures were beyond what our study could do while other measures were difficult to implement. For example, we knew that fear of pain could be effectively addressed by providing epidural analgesia, but some hospitals were constrained by anesthesia resources. Only 8 hospitals in our study provided epidural analgesia, resulting in still low epidural analgesia use (20–30% of women who attempted labor). Doula is effective in reducing CS use and increasing women’s satisfaction. But the overwhelming volume of deliveries in Chinese public hospitals hampers one-to-one doula or midwife support. In our study, only one third of parturients had doula. And a doula is often shared by more than one laboring woman.

We also knew that physicians play a critical role in CS decision making, prelabor, and intrapartum. But the “physician factor” is also complicated by multiple forces. The shortage of medical staff to handle the large volume of delivery, financial incentives, and constrained doctor-patient relationship all likely draw the decision leaning towards CS. Physicians’ practice pattern is often unclear. Our trial did not attempt to address the physician factor directly, which may be an important determinant in the success of an intervention trial on reducing the CS rate. For future studies, an assessment of practice pattern among physicians may provide useful insights. For example, asking the surgeon to complete a detailed reporting form that uncovers the indications as well as intentions and actions of the surgeon may help to identify potential targets for intervention. Given its importance, the physician factor could be a focus for future research. But the government health policy that can address some of the above issues may be more effective.

## Conclusions

Our multifaceted intervention for 8 months was not effective in reducing the CS rate in a large, multicenter cluster-randomized field trial in Shanghai, China. Further strategies that can be tailored to local contexts and drivers of CS are warranted to result in more effective measure to reduce the high CS rate. Government policy may have a greater impact on reducing the CS rate than the interventions initiated by hospitals or academic organizations.

## Supplementary information

Additional file 1: **Table S1.** The list of health education classes available to pregnant women.

Additional file 2. Study protocol.

Additional file 3: **Table S1.** CONSORT 2010 checklist of information to include when reporting a cluster randomised trial. **Table S2.** Extension of CONSORT for abstracts1,2 to reports of cluster randomised trials.

Additional file 4: Chart Abstraction Form for Maternal and Neonatal (or Stillbirth) Information.

## Data Availability

The datasets used and/or analyzed during the current study are available from the corresponding author on reasonable request.

## Abbreviations

ACOG: American College of Obstetricians and Gynecologists
ART: Assisted reproductive technology
BMI: Body mass index
CIs: Confidence intervals
CS: Cesarean section
GA: Gestational age
GEE: Generalized estimating equations
GWG: Gestational weight gain
ORs: Odds ratios
QuIPS: Quality Improvement and Patient Safety Committee
