# Supplementary material for: A cluster-randomized field trial to reduce cesarean section rates with a multifaceted intervention in Shanghai, China
Source: BMC Med. 2020 Feb 14;18:27. doi: 10.1186/s12916-020-1491-6 (PMC7020498; doi:10.1186/s12916-020-1491-6)
Supplement: Supplementary file 1 — Table S1. The list of health education classes available to pregnant women. [file 12916_2020_1491_MOESM1_ESM.docx]

**Table S1** The list of health education classes available to pregnant women

| **Course** | **Gestational weeks** | **Teaching method** |
| --- | --- | --- |
| Nutrition management in the first trimester | 10 | Online (live and video) |
| Happy labor | 12 | In-person class |
| Physical activities in early pregnancy | 14 | In-person class and online video |
| Prevention and treatment of pregnancy complications | 16 | In-person class |
| Nutrition management in the second and third trimester | 18 | Online (live, video) |
| Physical activities in second and third trimesters | 20 | In-person class and online video |
| Choice of mode of delivery | 22 | Online (live, video) |
| Labor analgesia | 24 | Online (live) |
| Breastfeeding guidance | 26 | Online (live, video) |
| Key issues in postpartum care | 28 | Online (live, video) |
| Lamaze pain reduction | 30 | In-person class and online video |
| Newborn care | 32 | Online (live) |
| Postpartum rehabilitation | 34 | Online (live) |
| Choice of mode of delivery | 36 | Online video |
| Newborn touching and bathing | Postpartum | Online video |
