# Supplementary material for: A cluster-randomized field trial to reduce cesarean section rates with a multifaceted intervention in Shanghai, China
Source: BMC Med. 2020 Feb 14;18:27. doi: 10.1186/s12916-020-1491-6 (PMC7020498; doi:10.1186/s12916-020-1491-6)
Supplement: Supplementary file 2 — Study protocol. [file 12916_2020_1491_MOESM2_ESM.docx]

**A Cluster-randomized Field Trial to Reduce Cesarean Section Rates and Improve the Quality of Newborn Population**

**NID Number:**

Clinical Trial Registration Number:

**Principal Investigator:**

Jun Zhang, Xinhua Hospital Affiliated to Shanghai Jiao Tong University School of Medicine

Liping Zhu, Shanghai Maternal and Child Health Center

**Project Team Member:**

Fei Dai, Shanghai Jiao Tong University School of Public Health

Rong Zhang, Shanghai Maternal and Child Health Center

Guohong Li, Shanghai Jiao Tong University School of Public Health

Hongying Dai, Shanghai University of Medicine & Health Sciences

Min Qin, Shanghai Maternal and Child Health Center

Luming Zou, Xinhua Hospital Affiliated to Shanghai Jiao Tong University School of Medicine

File Version: July 07, 2016

**Contents**

[Section 1. Abstract 3](#_Toc459962449)

[Section 2. Background and rationale 6](#_Toc459962450)

[2.1 Study hypothesis 6](#_Toc459962451)

[2.2 Background 6](#_Toc459962452)

[Section 3. Study design 12](#_Toc459962453)

[3.1 Primary OUTCOME 12](#_Toc459962454)

[3.2 Secondary OUTCOME 12](#_Toc459962455)

[3.3 Study interventions 12](#_Toc459962456)

[3.4 Blinding METHOD 12](#_Toc459962457)

[3.5 Potential risks and benefits 13](#_Toc459962458)

[Section 4. Study procedures 14](#_Toc459962459)

[4.1 Baseline survey 14](#_Toc459962460)

[4.2 Formulate MULTIFACED intervention TO REDUCE CS rate in Shanghai 14](#_Toc459962461)

[4.3 Monitoring of CS rate and maternal and child health evaluation 20](#_Toc459962462)

[Section 5. Data analysis 21](#_Toc459962463)

[5.1 Sample size and statistical power 21](#_Toc459962464)

[5.2 Statistical analysis method 22](#_Toc459962465)

[Section 6. Ethical considerations 23](#_Toc459962466)

[Section 7. References 24](#_Toc459962467)

[Appendix 1 26](#_Toc459962468)

# Section 1. Abstract

**Introduction**

Cesarean section (CS) rate in China has increased dramatically in recent years. In 2010, the World Health Organization (WHO) published a study in the Lancet, indicating that the overall CS rate was 27.3% and the assisted vaginal delivery rate was 3.2% in a sampling survey conducted among 9 Asian countries from October 2007 to May 2008. China had the highest CS rate of 46.2%, a rate that was more than 3 times of the upper limit recommended by WHO, among which 11.7% was without medical indications. Our previous survey found that the CS rate is up to 56% in Southeast China, with a non-medical indication CS rate of 20-22%. About 16 million neonates are born every year in China, which means that about 9 million of them are born by CS and up to 3 million are born by CS without any medical indication. Epidemiological studies have suggested that CS may increase the risk of asthma, allergic dermatitis, food allergy, attention deficit hyperactivity disorder and obesity in children. Therefore, controlling the CS rate and reducing unnecessary CS are important in primary prevention of some common diseases and highly prevalent disorders of children.

**Objectives**

1. Improve hospital management mode and construct feasible and comprehensive intervention measures by prenatal health education, improved hospital CS policy, and training of doulas and midwives;
2. Reduce the CS rate in Shanghai, especially the CS without medical indication.

**Study design**

This is a cluster-randomized field trial. Hospitals are divided into two groups randomly, with 10 hospitals each in the intervention and the control group.

**Inclusion criteria**

1. **Study sites selection:** 8 tertiary hospitals (including 2 maternity hospitals, 2 referral centers for critically ill obstetric patients, and 4 general hospitals) and 12 secondary hospitals (including 4 maternity hospitals and 8 general hospitals) in Shanghai will be selected as study sites.
2. **Study subjects**
   1. Subjects in the baseline survey are parturient who give birth in 20 participating hospitals from January 1, 2016 to June 30, 2016;
   2. Subjects in evaluation survey are parturients who give birth in 20 participating hospitals from January 1, 2017 to June 30, 2017;
   3. Inclusion criteria: Pregnant women who plan to give birth in any of the participating hospitals in this study.

**Stratification**

We stratify the hospitals into two strata according to the level of hospitals. The tertiary hospitals and secondary hospitals are divided into two groups respectively at a ratio of 1:1 by cluster randomization, with 10 hospitals each in the intervention and the control groups.

**Exclusion criteria**

None.

**Intervention**

From August 1, 2016, a multifaceted intervention targeting the major social factors which contribute to the high CS rate in China will be implemented, especially for those who prefer to give birth by CS without medical indication in the intervention hospitals. Targeted health education to pregnant women includes publicizing scientific knowledge of pregnancy and delivery to improve women’s correct understanding of the benefits and risks of CS. Training of midwives/doula includes improving the skills of midwives and doulas in the intervention hospital for better doula accompanied delivery. Improved hospital CS policy to lower CS rate will be formulated on the basis of the pilot survey and interviews and implemented as hospital management mode.

**Primary outcome**

The primary outcome is to reduce the CS rate, especially those without medical indication. High CS rate is one of the major challenges in maternal and child healthcare in Shanghai and across China. CS has various adverse effects on maternal and children’s health. This program aims to formulate a health education manual targeting both healthcare professionals and pregnant women/the public. It is expected to establish an effective model for reducing CS rate, which can be generalized to the healthcare facilities in Shanghai and even nationwide.

**Secondary outcome**

The secondary outcome includes gestational weight gain, maternal and neonatal complications, the average length of stay and the economic burden of the patients. Additionally, we will also perform systemic economic evaluation for the health policies and medical practices; perform analysis for the possibility of various risks of CS and the related cost effectiveness; perform comprehensive analysis for the social and economic benefits by reducing CS rate; and perform cost effectiveness analysis.

**Conclusion**

The CS rate has increased dramatically in many parts of the world. In this project, we carry out multifaceted intervention targeting the major social factors which contribute to the high CS rate in China. We assume that the multifaceted intervention can effectively reduce CS rate, especially the CS without medical indication, and improve the quality of newborn population.

# Section 2. Background and rationale

## 2.1 Study hypothesis

CS rate in China has increased dramatically in recent years. China had a highest CS rate of 46.2% over the world, a rate of more than 3 times of the upper limit recommended by WHO, among which 11.7% was without medical indications. Epidemiological studies have suggested that CS may increase the risk of asthma, allergic dermatitis, food allergy, attention deficit hyperactivity disorder and obesity in children. Therefore, controlling the CS rate and reducing the unnecessary CS are important in primary prevention of some common diseases and highly prevalent disorders of children.

We assume that the multifaceted intervention, including more targeted health education to pregnant women, improved hospital CS policy, and training of midwives/doula, can effectively decrease the CS rate, improve maternal and child health, and improve the quality of newborn population.

## 2.2 Background

**2.2.1. Causes of high CS rate in China and the benefits of natural childbirth**

CS rate has increased dramatically in China in recent years. A recent review published by the *British Journal of Obstetrics and Gynaecology* (BJOG 2014;8:1-5) comprehensively summarized the risk factors for high CS rate in China and grouped them into the following three categories:

- **Maternal healthcare system factors:** increased hospital deliveries, urbanization, doctors taking the place of midwives to deliver babies, the large volume of deliveries often lead to more convenient and scheduled CS;
- **Doctor/hospital factors:** the shortage of nurses/midwives, higher financial incentives for CS, the constrained doctor-patient relationship, loose indications for CS;
- **Patient factors:** single-child policy makes women not caring about subsequent pregnancy risk, excessive pursuit of perfect baby, elective CS due to traditional belief and fear of labor pain.

However, CS is only a means to manage difficult labor and maternal and neonatal complications, while the childbirth is a natural physiological process. Studies have shown that children delivered by natural childbirth may have an advantage than those delivered by CS in terms of cognition, health and other aspects. The uterus contracts regularly during the process of childbirth, which is benefit for the fetus to exercise his/her lungs and establish spontaneous breathing after birth and promoting the maturation of the lung. The uterine contraction and compression of birth canal during labor can expel out the amniotic fluid and mucus in the respiratory tract of the fetus, which can reduce the incidence of neonatal aspiration pneumonia and wet lung disease. Immunoglobulin G (IgG) can be transferred from mother to the fetus during natural childbirth. Children delivered by natural childbirth has lowered risk of allergy, respiratory tract infection, childhood obesity in later life than those delivered by CS, due to earlier exposure to natural bacterial flora and develop normal gastrointestinal flora. For parturient, natural childbirth has the following advantages: 1. Labor pains during natural vaginal delivery are associated with changes of uterus including thinning of lower segment of uterus, thickening of upper segment, and expansion of uterine orifice. These changes enable the parturient to have strong uterine contraction after childbirth, which is conducive to discharge of postpartum lochia, and uterine recovery. 2. Parturient has lower risk of bleeding, infection, adhesion and endometriosis due to lack of surgical wound on the abdominal wall. Therefore, parturient can recover more rapidly after delivery. 3. Doula accompany labor and labor analgesia can alleviate the labor pain. 4. Less expenditure due to shorter length of stay.

**2.2.2. Possible measures to lower CS rate**

For the increasing CS rate year by year, researchers at home and abroad have conducted numerous studies regarding the interventions to lower CS rate. The interventions include the following three aspects:

- **Health education**

The CS rate remains at a high level in China. In addition to the unavoidable health concerns, most CS are performed due to the fact that parturient are unfamiliar with natural childbirth and fear of labor pain. Therefore, prenatal health education is particularly important.

Health education includes the following aspects. Firstly, from the perspective of children, the pregnant women should learn the fact that only natural vaginal delivery can enable the baby to have more exercises and better immunity. Secondly, from the perspective of parturient, they should learn the fact that natural vaginal delivery will enable them have better experience of childbirth and rapid postpartum recovery. Lastly, the education should help parturient to overcome the fear of labor pain. In conclusion, health education aims to make parturient fully understand the basic process and the potential benefits of natural childbirth. Adequate scientific knowledge will help parturient overcome the feeling of fear and allow more women to choose natural vaginal delivery.

In addition, gestational weight gain is a common problem in China. A recent study ^[1]^ showed that the gestational weight gain among Chinese women is much earlier and higher than that of other countries (Ismail et al. BMJ 2016; 352: i555). Excessive weight gain during pregnancy increases not only the incidence of macrosomia, but also the risk of gestational diabetes mellitus, both of which will contribute to higher CS rate. Therefore, controlling gestational weight gain is an indirect method to reduce CS rate.


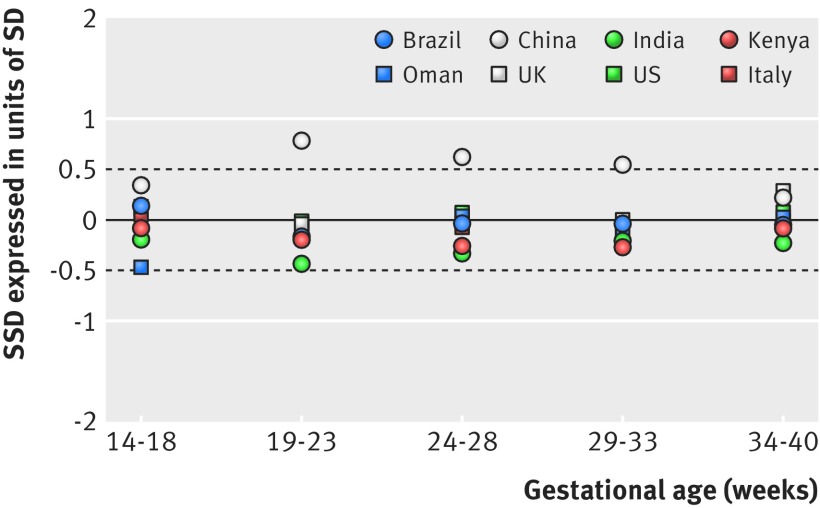


- **Doula support**

Numerous studies have shown that Doula support and improved midwifery skills can reduce CS rate. Doula means that an experienced woman provides pregnant women with consistent physical and emotional support during childbirth ^[2]^. Guadalupe Trueba, et al ^[3]^ found that Doula support during childbirth can not only significantly reduce CS rate and the use of oxytocin, but also bring positive physical, emotional and economic effects. This study also suggested that Doula support can shorten labor process, reduce the use of CS and epidural anesthesia, and decrease the requirement of oxytocin and forceps. Doula support during childbirth can relieve the labor pain and anxiety for parturient. On the other hand, Doula support can help parturient extend breastfeeding duration, and then reduce the incidence of neonatal vomiting, common colds, diarrhea, cough, loss of appetite and other problems. Additionally, Doula support can also strengthen the relationship between mother and child. Hodnett, et al ^[4]^ reported a higher satisfaction about the childbirth experience among women with Doula support during childbirth. Moreover, Doula can also reduce economic cost. Rachel Pilliod1, et al ^[5]^ reported that despite the low cost for every Doula support of less than 159.73 dollars, Doula support can reduce the use of obstetrical intervention and the risk of admission to neonatal intensive care unit (NICU).

- **Health policy**

Many countries have made bold reform and attempts regarding hospital management mode and health policy, implemented a lot of new hospital management modes and health policies, and achieved significant results in reducing CS rate. These measures primarily include the following aspects:

- - **Government policy**

Mandatory government policies are very effective in reducing CS rate. William E, et al ^[6]^ reported a decrease in CS rate by 90% within seven years in a quality incentive program for inpatients in Arkansas State. This program primarily imposed financial penalty for elective CS before 39 weeks of gestation.

- - **Hospital management**

In 2007, Canadian scholar Chaillet, et al ^[7]^ indicated that audit and feedback, quality improvement, and multifaceted strategies were effective for reducing CS rate. Moreover, multifaceted strategies, based on audit and detailed feedback, can improve clinical practice and effectively reduce CS rates.

- - - **Audit and feedback**

*Obstetricians must accept mandatory second opinion before CS*: In 2004, a study by Fernando Althabe, et al ^[8]^ in THE LANCET indicating that the mandatory second opinion policy reduced CS by 7.3%, most of which were intrapartum CS.

*Peer review including prenatal consultation before CS and postpartum surveillance*: Liang WH^[9]^ suggested that a weekly departmental cesarean indication conference should be hold to discuss every case of CS. The opinion of consultants must be solicited for all potential CS cases. The pregnant women are encouraged to have a trial of vaginal birth after cesarean. The feedback is provided in the form of a conference.

- - - **Quality improvement**

Active labor management and quality improvement are very important for reducing CS rate. EDRO A, et al ^[10]^ found that compared to service improvements and detailed feedback, the willingness of attending physician is more important to lower CS rate. In 2014, Joyce L ^[11]^ evaluated the *Focus on Normal Birth and Reducing Caesarean Section Rates Rapid Improvement Programme* in Yorkshire, England. The effective methods for reducing CS rate in this programme included priority of natural vaginal delivery, clear communication across disciplines and strong leadership, executive support and clinical leaders within each discipline. Finally, the programme reduced CS rate by 0.5% on average (from 26.4% to 25.9%).

- - **Role of professional society**

External peer review is also very effective for reducing CS rate. Bickell NA, et al ^[12]^ found that external peer review could reduce CS rate. External peer review is executed by a team trained by the American College of Obstetricians and Gynecologists. The team consists of four doctors and nurses. They visited the participating hospitals, interviewed key hospital staffs, and reviewed 100 delivery records to evaluate nursing quality. Eventually, the team provided feedback to the hospitals via talks, summary report or proposals.

- **Health economics**

CS is a relatively major surgical operation. The incidence of various accidents during and after the operation is much higher than natural vaginal delivery. Moreover, parturient with CS always has a longer average length of stay due to slow postpartum recovery. In the United States, CS is associated with an average hospital stay of 1.27 days longer than natural vaginal delivery. Traynor ^[13]^ reported that length of stay for parturient with natural vaginal delivery reduced by 31% than elective CS. Yu Yang ^[14]^ found that the average length of stay was 9.99 days for parturient with CS, while it was 7.14 days for natural vaginal delivery in Guangzhou, China. Length of stay for women with natural vaginal delivery reduced by 1.75-2.85 days.

Parturient with CS has longer length of stay, higher medical cost, more medical consumption for healthcare conditions, equipment, medicines and nursing services. Therefore, the medical expense for CS is significantly higher than that of natural vaginal delivery. Clark ^[15]^ and Henderson ^[16]^ reported that the medical expense for CS is 3.09 times and 1.94-2.74 times than natural vaginal delivery, respectively. Traynor ^[13]^ found that the medical expense for elective CS was 1.17 times than natural vaginal delivery. The difference in medical expense is mainly due to different length of stay and cost of operating room. In China, the average medical expense for CS is 1800 RMB higher than natural vaginal delivery, with an overall difference of 1274-1872 RMB and a daily reduction of 75-143 RMB in Guangzhou.

Parturient and their family may ask for CS for being fear of the potential accidents by natural vaginal delivery. Despite an increase of CS indicated by social factors, it still lack cost effectiveness study examining the potential risk of CS. Most of the existing studies only consider the short-term costs of superficial healthcare services. However, studies on the potential economic burden of the medium and long-term potential adverse consequences associated with CS are sparse. Reducing CS rate can shorten the length of stay and lower medical expense, which will save many medical resources.

In conclusion, it is an emerging trend worldwide to reduce CS rate by a multifaceted intervention including more targeted prenatal health education, improved hospital CS policy, and training of doulas and midwives. This project aimed to evaluate whether a multifaceted intervention targeting pregnant women, hospital policy and midwives/doula, would decrease the CS rate in China, improve maternal and child health, and improve the quality of newborn population.

**2.2.3. Effect of CS on children’s health**

In recent years, the CS rate remains at a high level in China. However, little is known about the long-term effect of CS on children’s health. Epidemiological studies had suggested that CS may increase the risk of allergic diseases such as asthma, allergic dermatitis and food allergy in children ^[17]^. A Norwegian maternal and child cohort study in 2011 found that CS was associated with an increased risk of asthma in children (relative risk=1.17, 95% CI: 1.03-1.32), suggesting a potential association between CS and atopic reactions ^[18]^. A recent meta-analysis revealed that CS increased the risk of asthma by 22% (OR = 1.22, 95% CI: 1.14, 1.29) ^[19]^. A nation-wide survey of asthma in 2010 by the National Pediatric Asthma Collaborative Group showed that the prevalence of asthma was 7.8% in children in Shanghai area, with an increase of 280% during the 10-year period from 1.97% in 2000 ^[20]^. Allergic diseases seriously affect the physical and mental health of children. Therefore, it has drew worldwide attention for the impact of CS on children’s allergic diseases. A survey in 2000 evaluated the effect of CS on maternal and children’s health in China. The infants were followed up to 1 year after birth. The results showed that the risk of diarrhea in infants delivered by CS was 1.25 times than infants by natural vaginal delivery (95% CI 1.01-1.56) ^[21]^. Therefore, this study suggested a close relationship between CS and allergic diseases, for the reason that food allergy may cause infantile diarrhea. In conclusion, this programme aims to evaluate whether a multifaceted intervention to reduce CS rate would improve the quality of newborn population. Various health outcomes, including the feeding profile, psychological and behavioral development, as well as the risk of food allergy, atopic dermatitis and other common allergic diseases, were evaluated in participating hospitals when following up the children to one year old after implementation of multifaceted intervention.

| Disease | Prevalence | OR (95% CI) |
| --- | --- | --- |
| Bronchial asthma | 7.57% ^a^ | 1.22 (1.14 - 1.29) |
| Allergic dermatitis /eczema | 8.3% ^b^ | 1.03 (0.98 - 1.09)  5.09 (1.57 – 16.5) |
| Allergic rhinitis | 14.9% ^c^ | 1.23 (1.12 - 1.35) |
| Food allergy | 6.2% ^d^ | 1.32 (1.12 - 1.55) |
| Obesity | 4.3% ^e^ | 1.68 (1.10-2.58) |

# Section 3. Study design

## 3.1 Primary outcome

The primary purpose of this study is to reduce the CS rate, especially the CS rate without medical indication in Shanghai. This program aims to formulate a health education measure targeting both healthcare professionals and pregnant women/the public. It is expected to establish an effective model for reducing CS rate, which can be generalized to the healthcare facilities in Shanghai and even nationwide.

## 3.2 Secondary outcome

The secondary outcome includes gestational weight gain, maternal and neonatal complications, the average length of stay and the economic burden of the patients. Additionally, we will also perform systemic economic evaluation for the health policies and medical practices; perform analysis for the possibility of various risks of CS and the related cost effectiveness; perform comprehensive analysis for the social and economic benefits by reducing CS rate; and perform cost effectiveness analysis.

## 3.3 Study interventions

From August 1, 2016, a multifaceted intervention targeting the major social factors which contribute to the high CS rate in China will be implemented targeting the pregnant women, especially those who prefer to give birth by CS without medical indication in the intervention hospital. Targeted health education to pregnant women includes publicizing scientific knowledge of pregnancy and delivery to improve women’s correct understanding of the benefits and risks of CS. Training of midwives/doula includes improving the skills of midwives and doulas in the intervention hospital for better doula accompanied delivery. Improved hospital CS policy to lower CS rate will be formulated on the basis of the pilot survey and interviews and implemented as hospital management mode.

## 3.4 Blinding method

No blinding method will be applied in this project.

## 3.5 Potential risks and benefits

The project involves a wide range and large number of study subjects, which requires close cooperation among participating hospitals. Therefore, adequate quality control is necessary. Lacking of data quality control is a potential risk in this study.

The project will implement multifaced interventions targeting patients, healthcare professionals and hospital management to reduce CS rate from different perspectives of “supply” and “demands” and CS management system. This study will also aim to formulate a large population-based intervention mode to reduce the high CS rate in China, and provide evidence for further improvement and implementation in Shanghai and across China.

# Section 4. Study procedures

## 4.1 Baseline survey

- - 1. **Study subjects**
- **Study site selection:** 8 tertiary hospitals (including 2 specialized hospitals for women and children, 2 consultation and rescue centers for critically ill obstetric patients, and 4 general hospitals) and 12 secondary hospitals (including 4 specialized hospitals and 8 general hospitals) in Shanghai will be selected as study sites.
- **Study subjects:**
  - Subjects in baseline survey are parturient who give birth in 20 participating hospitals from January 1, 2016 to June 30, 2016;
  - Subjects in evaluation survey are parturient who give birth in 20 participating hospitals from January 1, 2017 to June 30, 2017;
- **Inclusion criteria:** The pregnant women who plan to give birth in any of the participating hospitals in this study.

**4.1.2 Survey contents:** Medical records of mothers and newborns were retrieved and information on maternal demographic characteristics, reproductive history, medical history, details and complications of the current pregnancy, evaluation on admission, childbirth summary, and intrapartum and postpartum pathological conditions, as well as neonatal conditions were abstracted by specially trained research staff for both the baseline and evaluation surveys.

## 4.2 Formulate multifaced intervention to reduce CS rate in Shanghai

From August 1, 2016, multifaceted intervention targeting the major social factors which contribute to the high CS rate in China will be implemented targeting the pregnant women, especially those who prefer to give birth by CS without medical indication in the intervention hospital. Targeted health education to pregnant women includes publicizing scientific knowledge of pregnancy and delivery to improve women’s correct understanding of the benefits and risks of CS. Training of midwives/doula includes improving the skills of midwives and doulas in the intervention hospital for better doula accompanied delivery. Improved hospital CS policy to lower CS rate will be formulated on the basis of the pilot survey and interviews and implemented as hospital management mode.

**4.2.1 Health education**

**4.2.1.1 Preliminary preparation**

All the preparatory work must be finished before June 10, 2016 to ensure the feasibility of the project.

- - To build a database for the subjects (We can call it **Large Table** for short, which will be completed by the pregnant women themselves online via an APP):

This table will be kept by the study team. Every subject in the pilot intervention will have one table as the basis of evaluation, containing the following:

(1) Basic information of the subject to be intervened;

(2) The content, time and effect of health education activities during the full course of intervention;

(3) Other relevant information required by this study.

- - **To set up an education curriculum schedule (We can call is Small Table for short):**

This small table will record the time, content and effects of prenatal education intervention. This table can be designed as label records (labels are attached on the outer pages of back cover of the small medical card of the pregnant woman) or stamp record (stamp on the outer pages of back cover of the small medical card of the pregnant woman). This table is carried by the subjects themselves and presented when the subjects attending health education activities. The study staff will record the details of the activity situation in the table.

- - **Organize an expert panel:**

The project team and Center for Mother and Children Healthcare are responsible for selecting administrative staff, experts and relevant perinatal workers from the participating hospitals and institutes

- - **Set up a lecturer team:**

The project team and Center for Mother and Children Healthcare are responsible for selecting qualified high-level lecturers from the hospitals in Shanghai. The lecturers from the 10 hospitals participating in pilot intervention form a lecturer team. It is also appropriate for any qualified candidate to apply for this position as a lecturer.

- - **Set up a work group:**

The project team, Center for Mother and Children Healthcare, and a third-party internet company will form a work group to be responsible for arranging relevant events, such as make an appointment, organize and record for a meeting, activity, or educational course, contact, archiving of documents, as well as on-site communication, coordination and management, and so on.

- - **Prepare teaching materials and teaching aids:**

**Types of teaching materials:** leaflets, topic presentations, video clips, publications, textbook or handbook;

**Contents of teaching materials:** childbirth, nutrition and exercises or sports (weight management), psychological development, Lamaze method of childbirth, puerperium care, breastfeeding, neonatal care;

**Teaching aids used in intervention:** dummy (model) pelvis, dolls, nutritional models, breastfeeding models, as well as the environmental setting and layout of an experience hall (health education site) for demonstration and training of intervention measures.

**4.2.1.2 Protocol for implementation**

Maternal education plays an active role in controlling gestational complications, and avoiding unnecessary CS due to social factors or that without medical indication. The methods of health education intervention were described in Appendix. The specific measures include the following 4 aspects:

- **Community and maternity hospital**

Enrolled the subjects when she takes her “**small card (brief record)**” to the community. Then she will go to the maternity hospital to have her “**big card (full record)**” created. From the time when her big card is created to the expected date of delivery (when she waits for childbirth in hospital), the subjects’ gestation is during 6-36 weeks. During this period (about 30 weeks), the project team can provide various forms of prenatal education and training. The educational sessions must be provided in scheduled time at specified site, and predefined contents in various forms. The events should be organized carefully to make it interesting and fruitful for the subjects. The subjects are encouraged to actively participate in such activities and communicate with project team to meet the requirements of final evaluation.

- **Starting steps of intervention**

**Community:** When a pregnant woman takes her small card to the community, a community worker will introduce this study project to her, and complete the questionnaire, and communicate with the subject to obtain maternal informed consent. After the pregnant woman expresses that she voluntarily takes part in the intervention group of this study project, her personal archive will be created, coded by scanning, and stamped (i.e., the large table and small table). Then the community worker will make appointments (time, venue) with the subject for her educational events (childbirth, weight management) at district center for maternal and child healthcare. This represents the start of early intervention.

**Maternity hospital:** Roll-up banner or screen will be set in the obstetric clinic for the purpose to introduce the study project when the pregnant women came to create their pregnancy cards. If pregnant woman is willing to participate in the project, her archive will be coded by scanning. The teaching schedule will be provided online. The subject will be followed up in time to reserve the educational events for her. The medium-term intervention starts from this time.

- **Intervention of labor skills:**

For the purpose to enhance the confidence of pregnant women more effectively in normal birth, and reducing their fear of labor process, clinicians must contact with the pregnant woman face to face. A midwife will meet with the subjects participating in the intervention study during the period from December 2016 to March 2017. They will teach labor skills to the pregnant women and share some actual cases of natural vaginal delivery using their rich practical experience. They will also ask about the weight management situation during pregnancy.

- **Internet platform:**

Internet-based applications are used widely at present. In addition to the conventional teaching approach, the series of maternal education events can also be available online (E-learning), which enables the subjects to link more closely to the project team. The subject will be coded by scanning and enrolled in the online group maintained by the project team. The subjects can enjoy weekly feeds of maternal healthcare information, set their own interaction platform, and learn WeChat lessons.

**4.2.2 Examine the hospital management model favorable for reducing CS rate**

**4.2.2.1 Field survey**

Field survey will be conducted to understand the issues and challenges the healthcare providers are facing in reducing CS, especially what can be improved in hospital management model.

**4.2.2.2 Focus group interview**

Focus group interview will be organized for hospital management, department head, physicians, nurses, and midwifes to further understand the issues and challenges different healthcare workers in the medial institutions are facing in reducing CS rate. They will be asked to recommend some effective measures in hospital management model.

**4.2.2.3 Formulate intervention policies for reducing CS rate**

On the basis of review and summarization, efforts will be made to seek the support of policy-making department to draft the tentative management plan as intervention measures.

**4.2.2.4 Implementation of intervention**

A stratified random sampling method will be used to select 8 tertiary hospitals (including 2 maternity hospitals, 2 consultation and rescue centers for high-risk pregnant women, and 4 general hospitals) and 12 secondary hospitals (including 4 maternity hospitals and 8 general hospitals) in Shanghai. A total of 20 hospitals as study sites will be randomized into intervention group or control group to implement interventions in the intervention group.

**4.2.2.5 Outcome evaluation**

Outcome evaluation will be made for the intervention measures half to one year after initiation of intervention. Demographic characteristics, obstetric details, and economic information of the pregnant women who give birth in the participating hospitals will be collected as baseline data (baseline survey). The corresponding data will be collected once again half to one year after implementation of intervention policies to evaluate the effectiveness of intervention (evaluation survey). The parameters for evaluation include pregnancy outcome, quality of life and economic cost.

**4.2.3 Doula training**

**4.2.3.1 Study design**

- - - **Overall objective:** Doula intervention in the entire course of childbirth to support the parturient woman physically, psychologically, and emotionally. Doula will help and encourage the parturient woman to build confidence in normal birth. Doula midwifery will be promoted as a novel, innovative, scientific, ideal, and painless intrapartum service.
    - **Specific objective:** Appropriate Doula midwifery will help relieve labor pains, increase comfort, promote labor process, and make the parturient woman more efficient in delivery by herself, reduce episiotomy rate, and improve the positive childbirth experience. To suck the breast immediately after birth and early mother-infant contact will build up mother-child bondage, promote uterine contraction, and improve breastfeeding rate.
    - **Study procedures:** Doula training and provide Doula care and Doula midwifery services to the parturient women in clinical practice. Intervention measures are implemented to the parturient women in the 10 randomly sampled hospitals. According to specific individual needs, Doula care service and/or appropriate Doula midwifery techniques will be provided to make the parturient women complete their childbirth process comfortably, peacefully, successfully and naturally. These measures are expected to reduce CS rate, and effectively prevent the incidence of intrapartum complications.
    - **Study features and innovation:** Doula training, woman-oriented, consistent care, more focused on the psychological stability and humane midwifery, improve the Doula training plan from multiple dimensions and at multiple levels to make it completely suitable for Shanghai.

**4.2.3.2 Study contents**

- **Discussion and considerations:** Current status, personnel and financial issues of Doula midwifery in the hospitals in Shanghai.
- **Doula training:** training course, materials, lecturer team.
- **Candidates for Doula training:** obstetric staff in the 10 hospitals participating in the intervention study, including nurses and midwifes.
- **Clinical efficacy of Doula intervention:** improve positive childbirth experience, relieve anxiety of parturient women, satisfy the needs of pregnant women for childbirth knowledge, achieve humanized childbirth, reduce the CS due to social factors, and reduce episiotomy rate.
- **Study features:** “Doula” training, both Doula care and Doula midwifery, make efforts to solve the personnel issues, formulate Doula training plan and gradually improve to make it completely suitable for Shanghai.

**4.2.3.3 Implementation plan**

- **Consultation and analysis:** consultation, interview, Doula history and development, specific Doula events and experience in hospitals, qualification of Doula, preliminarily propose the name of Doula training workshop and the potential candidates of Doula. This will be completed at the beginning of March.
- **Prepare the table of contents of the training course:** organize core team members to discuss and draft the table of contents of the training course. The draft will be reviewed and evaluated by relevant experts, clinicians, and teaching staff. This will be completed by the end of March.
- **Prepare teaching course and teaching materials (handouts):** authors and contributors are organized to prepare the training course and teaching materials as required by the table of contents. (Some of the authors can be selected from the 10 participating hospitals). The draft manuscript will be completed between April and May. Two interim meetings will be held to discuss and review the manuscripts.
- **Lecturer team:** It would be better to select some of the authors of the training materials as lecturer. Foreign experts will also be invited as to give speech and lectures. The lecturer team will be formed in May.
- **Candidates for training (trainees):** The path and objective of the training will be specified at first. The training class will be organized and opened in mid-June. The first training workshop will enroll 30 participants (trainees). Priority is given to those working in the 10 participating hospitals. This job will be completed by the end of May.

## 4.3 Monitoring of CS rate and maternal and child health evaluation

The CS rate will be evaluated since January 1, 2017, six months after implementing comprehensive intervention. Cases of childbirth during the period from January 1, 2017 to June 30, 2017 will be retrieved from the 20 hospitals, reviewed and evaluated (evaluation survey) in details. The required data will be extracted to fill out the questionnaire. The parturient women will be asked to complete the basic demographic information and economic data. The primary objective (reduction of CS rate) will be tested after implementation of intervention measures, especially the CS without medical indication. Reduction of complication and health economic cost-benefits will also be evaluated.

# Section 5. Data analysis

## 5.1 Sample size and statistical power

**Calculation of sample size:** the cluster design is considered in the calculation.

Intra-cluster correlation coefficient $\rho$_I_: 0.011

Significance level **α**: 0.05

Statistical power: 1-β: 0.9

Baseline CS rate in Shanghai: p_0_ = 45%

Average sample size per cluster n_j_ = 500 subjects

Formula:
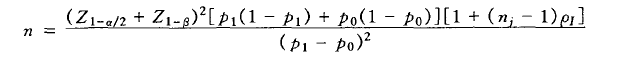


Number of clusters per treatment group: *N = n/n_j_*

If the CS rate decreases by 9% (i.e., from 45% to 36%), there should be 7 hospitals each in the intervention and the control group, with a total of about 17666 parturient. If the CS rate decreases by 6.75% (i.e., from 45% to 38.25%), there should be 12 hospitals each in the intervention and the control group, with a total of about 31788 parturient.

Twenty hospitals providing childbirth services will be selected from hospitals with an intention to participate in this project in Shanghai. We stratify the hospitals into two strata according to the level of hospitals. The tertiary hospitals and secondary hospitals are divided into two groups respectively at a ratio of 1:1 by cluster randomization, with 10 hospitals each in the intervention and the control group.

In each participating hospital, medical records of parturient who delivered during baseline survey (from January 1, 2016 to June 30, 2016) and evaluation survey (from January 1, 2017 to June 30, 2017) are randomly selected according to the hospital number, and the corresponding contents are extracted and the questionnaires are completed.

The sampling rule is defined as 40% for hospitals with 1000 to 3000 deliveries per year, 30% for hospitals with 3000-5000 deliveries per year, 20% for hospitals with 5000-9000 deliveries per year, and 10% for hospitals with 9000-15000 deliveries per year. It is estimated that each hospital will be selected about an average of 500-600 parturient. For parturient in the intervention group but don’t receive the health education intervention, we still regard them as intervention group based on intention-to-treat analysis.

## 5.2 Statistical analysis method

Continuous variables are described as mean (standard deviation), whereas categorical variables are presented as numbers and percentages. Generalized estimating equations (GEE) are used to assess the effects of the multifaceted intervention on CS rates and other secondary outcomes. All statistical analyses are conducted with SAS 9.4 software (SAS Institute Inc., Cary, NC).

# Section 6. Ethical considerations

Our Ethics board granted an approval for our study not to obtain individual patient’s informed consent due to midwife/doula training and hospital CS policy were unlikely to incur perceivable risks to the patients. We will post an informed consent form on an APP (Appendix 1) to ask whether a pregnant woman prefer to receive health education intervention. After learning the detailed information about the project and reading the informed consent form, the pregnant women will decide whether they want participate in the project by choosing agree or not agree option via the APP.

# Section 7. References

1. Cheikh Ismail L, Bishop DC, Pang R, Ohuma EO, Kac G, Abrams B, et al. Gestational weight gain standards based on women enrolled in the Fetal Growth Longitudinal Study of the INTERGROWTH-21st Project: a prospective longitudinal cohort study. BMJ. 2016;352:i555.
2. Doulas of North America (1992). Code of ethics and standards of practice. Seattle, WA: DONA.
3. Trueba G, Contreras C, Velazco MT, Lara EG, Martínez HB, et al. Alternative strategy to decrease cesarean section: support by doulas during labor. J Perinat Educ. 2000;9:8-13.
4. Hodnett, E. D. (1997). Support from caregivers during childbirth. In J. P. Neilson, C. A Crowther, E. D. Hodnett, G. J. Hofmeyer, & M. J. N. C. Keirse (Eds.), Pregnancy and childbirth module of the Cochrane database of systematic reviews.
5. Pilliod Doula care in active labor: a cost Benefit analysis. Supplement to JANUARY 2013 American Journal of Obstetrics & Gynecology S349.
6. Golden WE, Henry C, Palmer M. Reducing C-section rates in low-risk, first-time pregnancies. J Ark Med Soc. 2013;110:108-9.
7. Chaillet N, Dumont A. Evidence-based strategies for reducing cesarean section rates: a meta-analysis. Birth. 2007;34:53-64.
8. Althabe F, Belizán JM, Villar J, Alexander S, Bergel E, Ramos S, et al. Mandatory second opinion to reduce rates of unnecessary caesarean sections in Latin America: a cluster randomised controlled trial. Lancet. 2004;363:1934-40.
9. Liang WH, Yuan CC, Hung JH, Yang ML, Yang MJ, Chen YJ, et al. Effect of peer review and trial of labor on lowering cesarean section rates. J Chin Med Assoc. 2004;67:281-86.
10. Poma PA. Effect of departmental policies on cesarean delivery rates: a community hospital experience. Obstet Gynecol. 1998;91:1013-18.
11. Marshall JL, Spiby H, McCormick F. Evaluating the 'Focus on Normal Birth and Reducing Caesarean section Rates Rapid Improvement Programme': A mixed method study in England. Midwifery. 2015;31:332-40.
12. Bickell NA, Zdeb MS, Applegate MS, Roohan PJ, Sui AL. Effect of external peer review on cesarean delivery rates: a statewide program. Obstet Gynecol. 1996;87:664–67.
13. Traynor JD, Peaceman AM. Maternal hospital charges associated with trial of labor versus elective repeat cesarean section. Birth. 1998;25(2):81-84.
14. 于扬, 卢萍. 剖宫产与阴道分娩住院费用分析. 广东药学院学报. 2001;17:242-244.
15. Siassakos D, Clark J, Sibanda T, Attilakos G, Jefferys A, Cullen L, et al. A simple tool to measure patient perceptions of operative birth. BJOG. 2009;116:1755-61.
16. Henderson J, Petrou S. The economic case for planned cesarean section for breech presentation at term. CMAJ. 2006;174:1118-19.
17. Kozyrskyj AL, Bahreinian S, Azad MB. Early life exposures: impact on asthma and allergic disease. Curr Opin Allergy Clin Immunol. 2011;11(5):400-406.
18. Magnus MC, Håberg SE, Stigum H, Nafstad P, London SJ, Vangen S, et al. Delivery by Cesarean section and early childhood respiratory symptoms and disorders: the Norwegian mother and child cohort study. Am J Epidemiol. 2011;174:1275-85.
19. Thavagnanam S, Fleming J, Bromley A, Shields MD, Cardwell CR. A meta-analysis of the association between Caesarean section and childhood asthma. Clin Exp Allergy. 2008;38:629-33.
20. 陈育智. 中国城区儿童哮喘患病率调查. 中华儿科杂志. 2003; 42:123-123.
21. 梁红, 周利锋, 王炳顺, 周晔, 朱丽萍, 高尔升. 剖宫产分娩对婴儿健康影响的前瞻性研究. 生殖与避孕. 2007;27:124-127.

# Appendix 1

**WELCOMING WORDS**

**Dear mother-to-be,**

Welcome to attend the free lectures on maternal healthcare provided by Shanghai Maternal and Child Health Center. The lectures include online videos, in-person class, online live classes and expert counseling, aiming to make you access the standard and professional guidance and avoid misleading by partial information. We wish to achieve the goal: keep maternal and child safe and healthy, as well as improve the quality of newborn population!

(We will not promote or recommend any commercial product to you).

We will ask you several simple questions to help us understand your true intention and needs, and facilitate the successful progress of this project. We will also need your cooperation and support by answering these questions truthfully. We will strictly keep your information private and confidential.

A “Lucky Draw” activity is offered to you after the “questionnaire” is completed. We will tell you how and when to take the awards via mobile phone if you have a good luck.

Wish you and your baby healthy and happy! Thank you for your cooperation!

**Shanghai Maternal and Child Health Center**

**August 2016**

**The awards are VIP Obstetric Clinic Service:**

**A. Individualized nutrition counseling; B. Breastfeeding guide; C. Pelvic floor rehabilitation consultation; D. Pelvic floor rehabilitation (make appointment after labor).**

The award values about 300-10000 RMB. The gift coupon can be used immediately and is valid for 2 months. (Five winners for each service, but pregnant women do not know the value of their awards. Pregnant women are informed whether they need to make an appointment and the specific time and location. Time: every Thursday afternoon, 2:00 PM; Location: outpatient department at maternal and child healthcare center)

I agree to participate in this project

I do not agree to participate in this project
