# Supplementary material for: A cluster-randomized field trial to reduce cesarean section rates with a multifaceted intervention in Shanghai, China
Source: BMC Med. 2020 Feb 14;18:27. doi: 10.1186/s12916-020-1491-6 (PMC7020498; doi:10.1186/s12916-020-1491-6)
Supplement: Supplementary file 4 — Chart Abstraction Form for Maternal and Neonatal (or Stillbirth) Information. [file 12916_2020_1491_MOESM4_ESM.docx]

**Chart Abstraction Form for Maternal and Neonatal (or Stillbirth) Information**

**Inclusion criteria: gestational age ≥24 weeks+0 days, or birth weight ≥500g, including live birth, antepartum fetal death, intrapartum fetal death, and spontaneous abortion in second trimester**

**I. Maternal Demographics**

1. Date of chart abstraction 20|___|___| (year)|___|___| (month)|___|___| (day) unknow fill in 99

2. Woman’s name________________________ (on paper copy only, not entered into the database)

3. Hospital admission ID |___|___|___|___|___|___|___|___|___|___|___|___|___|___|___|___|___|

4. Women’s birth date |___|___|___|___| (year)|___|___| (month)|___|___| (day) unknow fill in 99

5. Women’s ID |___|___|___|___|___|___|___|___|___|___|___|___|___|___|___|___|___|___|

6. Woman’s address___________________________________________________

7. Education (1) illiteracy (2) primary school (3) secondary school (4) high school (technical school)

(5) junior college (6) college (7) graduate school (9) unkown

8. Work (1) employees (2) peasant (3) military (4) office Clerk

(5) science and technology personnel, medical personnel, teachers (6) entertainer

(7) student (8) unemployed (9) other (99) unkown

9. Payment method of medical costs

(1) basic medical insurance for urban employees (2) basic medical insurance for urban residents

(3) new rural cooperative medical system (4) poverty relief

(5) commercial health insurance (6) free medical care

(7) at one's own expense (8) medical insurance for urban and rural residents

(9) other social insurance

(10) other (e.g. maternity insurance, work-related injury insurance, migrant worker insurance, etc.)

(99) unknown

**II. Reproductive History**

1. Gravidity |___| unknow fill in 9 2. Parity |___| unknow fill in 9

3. Number of previous term birth |___| unknow fill in 9

4. Number of previous preterm birth |___| unknow fill in 9

5. Number of previous spontaneous abortion |___| unknow fill in 9

6. Number of previous induced abortion |___| unknow fill in 9

7. Number of previous stillbirth |___| unknow fill in 9

8. Number of previous cesarean delivery |___| unknow fill in 9

**III. Medical History**

1. Preexisting diabetes (1) yes (2) no (9) unknown 2. Heart disease (1) yes (2) no (9) unknown

3. Renal disease (1) yes (2) no (9) unknown 4. Autoimmune disorder (1) yes (2) no (9) unknown

5. Preexisting thyroid dysfunction

5.1. Hyperthyroidism: (1) yes (2) no (9) unknown

5.2. Hypothyroidism: (1) yes (2) no (9) unknown

5.3. Other thyroid dysfunction: (1) yes (2) no (9) unknown

**IV. Prenatal History of Current Pregnancy**

1. Gestational age at booking |___|___|week unknown fill in 99

2. Weight at booking |___|___|___|.|___|kg unknown fill in 999.9

3. Height |___|___|___|cm unknown fill in 999

4. Number of fetuses |___| unknown fill in 9

5. Spontaneous conception (1) yes (2) no (9) unknown

6. Thyroid disease

6.1. Hyperthyroidism: (1) yes (2) no (9) unknown

6.2. Hypothyroidism: (1) yes (2) no (9) unknown

6.3. Other thyroid disorders: (1) yes (2) no (9) unknown

7. Hypertensive disorders in pregnancy

(0) no

(1) gestational hypertension or pregnancy induced hypertension (PIH) (without proteinuria)

(2) mild preeclampsia (3) severe preeclampsia

(4) HELLP syndrome (5) eclampsia

(6) preeclampsia/eclampsia superimposed upon chronic hypertension

(7) chronic hypertension (chronic hypertension during this pregnancy (excludes gestational hypertension, preeclampsia or unspecified hypertension)

(8) unspecified hypertension (9)unknown

8. Gestational diabetes (1) yes (2) no (9) unknown

8.1. Use insulin to control gestational diabetes (1) yes (2) no (9) unknown

9. Placenta previa (1) yes (2) no (9) unknown

10. Abruptio placentae (1) yes (2) no (9) unknown

11. Premature rupture of the membranes (1) yes (2) no (9) unknown

12. Rh incompatibility (1) yes (2) no (9) unknown

13. Antepartum fetal death (1) yes (2) no (9) unknown

14. External cephalic version (1) yes (2) no (9) unknown

**V. Evaluation at Admission to Labor & Delivery**

1. Mode of pregnancy termination

(1) vaginal delivery (a. spontaneous labor, b. induced labor) (2) cesarean delivery

(3) induced abortion in second trimester (24-27 weeks) (4) spontaneous abortion (24-27 weeks) (9) unknown

2. Date and time of admission

20|___|___|(year)|___|___|(month)|___|___|(day) |___|___|:|___|___| (24 hours) unknown fill in 99

3. Best estimate of gestational age |___|___|week|___|day unknown fill in 99.9

4. Current weight |___|___|___|.|___|kg unknown fill in 999.9

5. Date and time of onset of spontaneous labor

20|___|___|(year)|___|___| (month)|___|___| (day) |___|___|:|___|___|(24 hours) unknown fill in 99

6. Frequency of uterine contraction |___|___|/10 minutes unknown fill in 99

7. Effacement |___|___|% or cervical canal length |___|.|___|cm unknown fill in 99

8. Cervical dilation |___|___|.|___|cm unknown fill in 99.9

9. Station (in 5ths) + or - |___| unknown fill in 9

10. Presentation (1) cephalic (2) breech (3) transverse lie (4) other (oblique / shoulder/ compound)

(9) unknown

**VI. Labor & Delivery Summary**

1. Onset of labor (1) spontaneous labor (2) labor induction (i.e. labor induction in third trimester)

(3) prelabor cesarean delivery (9) unknown

2. Labor induction (i.e. labor induction in third trimester) (1) yes (fill in 2.1-2.3) (2) no (9) unknown

2.1. The main indication for labor induction (Choose one)

(1) elective induction (no medical indication)

(2) late-term or postdate pregnancy (gestational age ≥41 weeks)

(3) maternal diseases, e.g. severe diabetes, hypertension, renal disease, etc

(4) premature rupture of the membranes

(5) fetal condition, e.g. suspected fetal distress, placental factors, suspected macrosomia, etc

(6) stillbirth or fetal anomaly

(7) other

(9) unknown

2.2. Method of labor induction (multiple selections allowed)

(1) aniotomy (2) msoprostol/PGE1 (3) mechanical (artificial rupture of membranes, balloon device, laminaria） (4) oxytocin (9) unknown

2.3. Date and time of labor induction

20|___|___|(year)|___|___| (month)|___|___| (day) |___|___|:|___|___|(24 hours) unknown fill in 99

3. Labor augmentation with oxytocin (1) yes (2) no (9) unknown

4. Doula accompanies delivery (1) yes (2) no (9) unknown

5. Method of rupture of membranes

(1) natural membrane rupture (2) aniotomy (3) rupture of membrane during cesarean section (9) unknown

6. Labor analgesia

(1) Medicine (a. epidural anesthesia b. spinal anesthesia (spinal anesthesia) c. spinal-epidural anesthesia

d. laughing gas)

(2) Non-medicine (a. Doula instrument b. labor ball c. water birth)

(3) none

(9) unknown

7. Date and time of complete cervical dilation

20|___|___|(year)|___|___| (month)|___|___| (day) |___|___|:|___|___|(24 hours) unknown fill in 99

8. Date and time of delivery of infant

20|___|___|(year)|___|___| (month)|___|___| (day) |___|___|:|___|___|(24 hours) unknown fill in 99

9. Date and time of delivery of placenta

20|___|___|(year)|___|___| (month)|___|___| (day) |___|___|:|___|___|(24 hours) unknown fill in 99

10. Meconium stain (1) yes (2) no (9) unknown

11. Placenta accrete/placenta increta (1) yes (2) no (9) unknown

12. Cord prolapse (1) yes (2) no (9) unknown

13. Fetal distress (1) yes (2) no (9) unknown

14. Shoulder dystocia (1) yes (2) no (9) unknown

15. Uterine rupture  (1) yes (2) no (9) unknown

16. Uterine dehiscence (1) yes (2) no (9) unknown

17. Fetal presentation at delivery

If single birth: (1) cephalic (2) breech (3) transverse lie (4) other (oblique/ shoulder/ compound)

(9) unknown

If multiple gestation: (1) cephalic-cephalic (2) cephalic-breech (3) breech-cephalic (4) breech-breech

(5) triplets or higher multiple gestation (9) unknown

18. Fetal position (1) OA (2) OP (3) OT (4) face (including brow) (5) other vertex (6) non-vertex

(9) unknown

19. Mode of delivery (1) spontaneous vaginal delivery (2) instrumental vaginal delivery (forceps, vacuum extraction)

(3) prelabor cesarean delivery (4) intrapartum cesarean delivery (9) unknown

20. Cesarean delivery

20.1. Indication for cesarean delivery

| **Indication** | **Major (Choose one )** | **Minor (Choose one** **)** |
| --- | --- | --- |
| 1. None, elective/request |  |  |
| 2. Previous uterine scar |  |  |
| 3. Breech/malpresentation |  |  |
| 4. Non-reasurring fetal testing/ fetal distress |  |  |
| 5. Failure to progress/ cephalopelvic disproportion |  |  |
| 6. Multiple gestation |  |  |
| 7. Meconium stain |  |  |
| 8. Fetal macrosomia |  |  |
| 9. Placenta previa or vasa previa |  |  |
| 10. Intrahepatic cholestasis of pregnancy |  |  |
| 11. Hypertensive disease, preeclampsia |  |  |
| 12. Failed induction |  |  |
| 13. Failed trial forceps or vacuum |  |  |
| 14. Placenta abruption |  |  |
| 15. Shoulder dystocia |  |  |
| 16. Fetal anomaly |  |  |
| 17. Chorioamnionitis |  |  |
| 18. Other emergency |  |  |
| 19. History of shoulder dystocia |  |  |
| 20. HIV, herpes active lesions |  |  |
| 21. Cephalopelvic disproportion |  |  |
| 22. Cord prolapse |  |  |
| 23. Diabetes |  |  |
| 24. Fetal growth restriction/ small for gestational age |  |  |
| 25. Autoimmune disorder |  |  |
| 26. Premature rupture of the membranes |  |  |
| 27. Heart disease |  |  |
| 28. Preterm birth |  |  |
| 29. Other fetal conditions |  |  |
| 30. Other maternal conditions |  |  |
| 99. Unknown |  |  |

20.2. Date and time when the operation began

20|___|___|(year)|___|___| (month)|___|___| (day) |___|___|:|___|___|(24 hours) unknown fill in 99

20.3. Date and time when the operation ended

20|___|___|(year)|___|___| (month)|___|___| (day) |___|___|:|___|___|(24 hours) unknown fill in 99

21. Episiotomy (1) midline (2) mediolateral (3) no (9) unknown

22. Hysterectomy (1) yes (2) no (9) unknown

23. Estimated blood loss (EBL) at time of delivery (1) <500 ml (2) 500–1000 ml (3) 1000-2000 ml (4) 2000-3000 ml (5) ≥3000ml (9) unknown

24. Blood products (1) yes (2) no (9) unknown

25. Delivery person (1) midwife (2) attending physician and above (3) resident (4) other (9) unknown

**VII. Maternal Intrapartum and Postpartum Condition**

1. Postpartum hemorrhage (1) yes (2) no (9) unknown

2. Perineal lacerations (1) I˚ (2) II˚ (3) III˚ (4) IV˚ (5) no (9) unknown

3. Blood transfusion (1) yes (2) no (9) unknown

4. Puerperal infection (1) yes (2) no (9) unknown

5. Ileus complications (1) yes (2) no (9) unknown

6. Amniotic Fluid Embolism (1) yes (2) no (9) unknown

7. Pulmonary Thromboembolism (1) yes (2) no (9) unknown

8. Thrombosis (1) yes (2) no (9) unknown

9. Maternal ICU admission (1) yes (2) no (9) unknown

10. Date of discharge

20|___|___|(year)|___|___| (month)|___|___| (day) unknown fill in 99

11. Maternal death (1) yes (2) no (9) unknown

**VIII. Newborn Information (one record for each baby)**

1. Date of birth 20|___|___|(year)|___|___|(month)|___|___|(day) unknown fill in 99

2. Mother’s hospital admission ID |___|___|___|___|___|___|___|___|___|___|___|___|___|___|___|___|___|

3. Plurality of gestation (1) singleton (2) twins (3) triplets or higher (9) unknown

4. Birth order (for multifetal gestation) ( Enter “0” for singleton) |___| unknown fill in 9

5. Birth outcome (1) livebirth (2) antepartum fetal death (3) intrapartum fetal death (4) neonatal death (9) unknown

6. Sex (1) male (2) female (3) ambigious (9) unknown

7. Birthweight |___|___|___|___|g unknown fill in 9999

8. 5 minute Apgar score |___|___| not evaluated fill in 88 unknown fill in 99

9. Birth injury (1) yes (2) no (9) unknown

10. Congenital anomalies (1) yes (2) no (9) unknown

If yes, provide values for all major and minor congenital anomalies:

____________________________________ __________________________________

_____________________________________ __________________________________

_____________________________________ __________________________________

11. NICU or neonatal ward admission (1) NICU admission (2) no (3) neonatal ward admission (9) unknown

12. Date of discharge or transfer 20|___|___|(year)|___|___|(month)|___|___|(day) unknown fill in 99

13. If the newborn was referred to another hospital, provide the hospital’s name _________________________________________

(Please acquire NICU or newborn outcomes)

**IX.** **NICU or neonatal ward Information (one record for each baby)**

1. Date of birth 20|___|___|(year)|___|___|(month)|___|___|(day) unknown fill in 99

2. Mother’s hospital admission ID |___|___|___|___|___|___|___|___|___|___|___|___|___|___|___|___|___|

3. Neonatal hospital admission ID |___|___|___|___|___|___|___|___|___|___|___|___|___|___|___|___|___|

4. Date of NICU adimission 20|___|___|(year)|___|___|(month)|___|___|(day) unknown fill in 99

5. Birth weight |___|___|___|___|g unknown fill in 9999

6. Respiratory distress syndrome (RDS) (1) yes (2) no (9) unknown

7. Pneumonia (1) yes (2) no (9) unknown

8. Intrapartum aspiration (1) yes (2) no (9) unknown

9. Apnea/bradycardia (1) yes (2) no (9) unknown

10. Neonatal asphyxia (1) yes (2) no (9) unknown

11. Seizure (1) yes (2) no (9) unknown

12. Renal failure (1) yes (2) no (9) unknown

13. Myocardiopathy (1) yes (2) no (9) unknown

14. Hypoxic-ischemic encephalopathy (1) yes (2) no (9) unknown

15. Periventricular-intraventricular hemorrhage (1) yes (2) no (9) unknown

16. Intracranial hemorrhage (1) yes (2) no (9) unknown

17. Periventricular leukomalacia (1) yes (2) no (9) unknown

18. Necrotizing enterocolitis (1) yes (2) no (9) unknown

19. Retinopathy of prematurity (1) yes (2) no (9) unknown

20. Sepsis (suspected, confirmed) (1) yes (2) no (9) unknown

21. Anemia (1) yes (2) no (9) unknown

22. Mechanical ventilator use (1) yes (2) no (9) unknown

23. Use of CPAP while in the NICU (1) yes (2) no (9) unknown

24. Transient tachypnea of the newborn (TTN) (1) yes (2) no (9) unknown

25. Exchange transfusion (treatment of jaundice) (1) yes (2) no (9) unknown

26. Twin-twin transfusion syndrome (1) yes (2) no (9) unknown

27. Date of discharge from NICU 20|___|___|(year)|___|___|(month)|___|___|(day) unknown fill in 99

28. Neonatal death (1) yes (2) no (9) unknown

29. Date and time of neonatal death

20|___|___|(year)|___|___|(month)|___|___|(day) |___|___|:|___|___|(24hours) unknown fill in 99

30. Cause of neonatal death

| Causes | Major (Choose one) | Minor (Choose one ) |
| --- | --- | --- |
| 1. Preterm birth/ low birth weight |  |  |
| 2. Congenital anomalies |  |  |
| 3. Asphyxia during the delivery/ HIE |  |  |
| 4. Aspiration syndrome of the newborn |  |  |
| 5. Neonatal infection |  |  |
| 6. Necrotizing enterocolitis |  |  |
| 7. Respiratory distress syndrome/hyaline membrane disease |  |  |
| 8. Pneumorrhagia |  |  |
| 9. Persistent pulmonary hypertension |  |  |
| 10. Hemolysis |  |  |
| 11. Electrolyte disturbances |  |  |
| 12. Accidental suffocation |  |  |
| 13. Other |  |  |
| 99. Unknown |  |  |
